# Histidine Kinase Sln1 and cAMP/PKA Signaling Pathways Antagonistically Regulate *Sporisorium scitamineum* Mating and Virulence via Transcription Factor Prf1

**DOI:** 10.3390/jof7080610

**Published:** 2021-07-28

**Authors:** Enping Cai, Shuquan Sun, Yizhen Deng, Peishen Huang, Xian Sun, Yuting Wang, Changqing Chang, Zide Jiang

**Affiliations:** 1 College of Plant Protection, South China Agricultural University, Guangzhou 510642, China; dlcep@foxmail.com (E.C.); sunshuquan@yrcti.edu.cn (S.S.); dengyz@scau.edu.cn (Y.D.); hps547384492@gmail.com (P.H.); sunx2021@163.com (X.S.); 2 Integrate Microbiology Research Center, Guangdong Province Key Laboratory of Microbial Signals and Disease Control, South China Agricultural University, Guangzhou 510642, China; yutingwbio@163.com; 3 Environmental Monitoring and Remediation Engineering Technology Research Center, School of Environmental Engineering, Yellow River Conservancy Technical Institute, Kaifeng 475004, China

**Keywords:** histidine kinase Sln1, cAMP/PKA, *Sporisorium scitamineum*, mating, cross-talk

## Abstract

Many prokaryotes and eukaryotes utilize two-component signaling pathways to counter environmental stress and regulate virulence genes associated with infection. In this study, we identified and characterized a conserved histidine kinase (SsSln1), which is the sensor of the two-component system of Sln1–Ypd1–Ssk1 in *Sporisorium scitamineum*. *SsSln1* null mutant exhibited enhanced mating and virulence capabilities in *S. scitamineum*, which is opposite to what has been reported in *Candida albicans*. Further investigations revealed that the deletion of *SsSLN1* enhanced SsHog1 phosphorylation and nuclear localization and thus promoted *S. scitamineum* mating. Interestingly, SsSln1 and cAMP/PKA signaling pathways antagonistically regulated the transcription of pheromone-responsive transcription factor SsPrf1, for regulating *S. scitamineum* mating and virulence. In short, the study depicts a novel mechanism in which the cross-talk between SsSln1 and cAMP/PKA pathways antagonistically regulates mating and virulence by balancing the transcription of the *SsPRF1* gene in *S. scitamineum*.

## 1. Introduction

The basidiomycetous fungus *Sporisorium scitamineum* is a global pathogen of sugarcane smut disease that causes substantial losses in cane yield and the sugar industry. *S. scitamineum*, as *Ustilago maydis*, is bipolar and undergoes three life stages, of haploid sporidium, yeast-like, non-pathogenic, dikaryotic hypha and diploid teliospore [1]. Haploid sporidia of two opposite mating types, *MAT-1* and *MAT-2*, can recognize each other to undergo fusion, a process known as sexual mating [2]. After sexual mating, the b locus encodes an active heterodimeric transcription factor complex composed of bE and bW proteins derived from different alleles to control filamentation in *S. scitamineum* [2]. The fusion of two haploid cells of opposite mating types is necessary to form invasively dikaryotic hyphae in *S. scitamineum* to infect the host [1]. Thus, mating/filamentation plays a key role in *S. scitamineum* pathogenicity.

The two-component phosphorelay system is widely found, which regulates a variety of cellular processes such as response to environmental stimuli, cell differentiation, secondary metabolite production, antibiotic resistance and virulence, in plant and animal pathogens [3,4]. The two-component phosphorelay system in most eukaryotes mainly consists of a sensor histidine kinase (HK) component, a His-containing phosphotransfer protein component and a response regulator (RR) component that transmits signals [5,6]. The two-component phosphorelay system in yeast is composed of Sln1–Ypd1–Ssk1 and a mitogen-activated protein kinase cascade consisting of Ssk2/Ssk22–Pbs2–Hog1 for the hypertonicity response through osmolarity signal transmission [7,8]. The histidine kinase (ortholog to Sln1) in filamentous fungi (*Aspergillus nidulans* and *Neurospora crassa*) also adapts to highly osmotic conditions by participating in the osmotic sensitive signal transduction pathway [9,10,11]. The lethality associated with the deletion of the *SLN1* or *YPD1* gene in *Saccharomyces cerevisiae* has highlighted this family of phosphate relay proteins as a potential prime antifungal target [12,13]. Three virulence-related conventional histidine kinase genes have been identified in *C. albicans* [14], whereas histidine kinase Sln1 in *Magnaporthe oryzae* senses and controls turgor-driven infection [6,15]. However, the two-component phosphorelay system among the smut fungi remains largely unknown.

The Hog1 activator has two key upstream osmotic response branches (*SLN1* and *SHO1* branches) for the high-osmolarity glycerol (HOG) pathway, which are redundant for cell survival under hyperosmosis [7,16,17,18]. However, the *SLN1* branch seems to be more important in pathway response to low or extremely high osmotic stress [19,20,21]. In the *SLN1* branch, the phosphorylated Sln1 triggers Ssk1 phosphorylation through intermediary phosphorelay protein Ypd1 [7], and Ssk1 phosphorylation further phosphorylates Ssk2/Ssk22 to transmit the signals [17,22]. In another branch, Sho1 senses hyperosmosis and activates the cytoplasmic MAPKKK Ste11 [23]. Finally, the activation of Ssk2 and Ste11 phosphorylates the MAPKK Pbs2, which in turn phosphorylates and activates the MAPK Hog1 [8,24]. Phosphorylation of Hog1 localizes in the nucleus, where the promoter region of the target gene(s) recruits it to activate several transcription factors [25,26,27,28]. Osm1 (ortholog to Hog1) activates Atf1 phosphorylation in *M. oryzae*, initiating the transcription of Ptp1/2 for the dephosphorylation of Osm1 [29]. The negative feedback loop of the Hog1 kinase was also noted in *Cryptococcus neoformans* [30].

The cAMP-dependent protein kinase A (PKA) signaling pathway is important and ubiquitously exists in eukaryotic organisms. This pathway participates in regulating physiological processes, such as morphological switch, response to stress, polarized cell growth, mating and virulence [31,32,33,34,35,36,37]. The core components of the cAMP/PKA pathway, such as G-protein, adenylyl cyclase and catalytic subunit of cAMP-dependent protein kinase A, contribute to mating and virulence in *S. scitamineum* [38]. The pheromone response factor SsPrf1, the downstream element of cAMP/PKA signaling, is also involved in the mating and virulence in *S. scitamineum* [39]. The interaction of the cAMP/PKA signaling pathway with other signal transduction pathways to exhibit a cross-talk relationship has been reported. For example, the Ime2-related protein encoded by *CRK1* acted as a signal integrator of external signals that were transmitted via cAMP/PKA and Kpp2–MAPK pathways in *U. maydis* [40]. Sln1 interacts with Sum1 in *M. oryzae*, which is a regulatory subunit of cAMP-dependent protein kinase A (PKA) [15]. However, the relationship between cAMP/PKA and SsSln1 in the smut fungi is unclear.

Therefore, during this study, Sln1 ortholog (SsSln1) was identified and characterized in *S. scitamineum* to understand its contribution in the regulation of virulence through triggering downstream signaling. The study revealed that the deletion of the *SsSLN1* gene enhanced the phosphorylation and nuclear localization of SsHog1. Further investigations depicted that the phosphorylation level of SsHog1 was essential for the mating and virulence of *S. scitamineum*. Intriguingly, we found that histidine kinase SsSln1 and cAMP/PKA pathways affected the transcription of *SsPRF1* gene to antagonistically regulate mating and virulence. In short, our results indicate that deletion of the *SsSLN1* gene enhances *S. scitamineum* mating and virulence capabilities, and reveal a cross-talk relationship between SsSln1 and the cAMP/PKA signaling pathway.

## 2. Materials and Methods

### 2.1. Strains and Growth Conditions

Two *S. scitamineum* wild-type haploid *MAT-1* (*a1 b1*) and *MAT-2* (*a2 b2*) were isolated and identified by Yan [2], and stored locally. The haploid sporidia were cultured on YePS liquid medium (yeast extract 1%, peptone 2%, sucrose 2%, pH 6.5) at 28 °C for 1–2 days as described [2,38]. For sexual mating assay, the fresh haploid sporidia were cultured in YePS medium to about O.D.600 = 1.0. The sporidia were collected and washed twice in sterilized double-distilled water (ddH_2_O), and the concentration was adjusted to O.D.600 = 1.0 with sterilized ddH_2_O. The haploid sporidia of opposite mating types were mixed at equal volume (O.D.600 = 1.0) and spotted on YePSA (YePS, containing 2% agar) or minimal medium [38], and allowed to incubate at 28 °C for 1–2 days.

### 2.2. Construction of Strains

The deletion, reintegration and overexpression of genes were performed by polyethylene glycol (PEG) mediate protoplast transformation using a split marker approach as described previously [38,41,42]. The Hygromycin resistance (*HYG^R^*) cassette was used as a resistance screening gene. The deletion mutants were generated by individually disrupting *SsSLN1* and *SsATF1* genes in the *MAT-1* and *MAT-2* strains. The left and right borders of *SsSLN1* and *SsATF1* genes were PCR amplified from *S. scitamineum* wild-type genomic DNA with the primers SsSLN1-LB-F/SsSLN1-LB-R, SsSLN1-RB-F/SsSLN1-RB-R, SsATF1-LB-F/SsATF1-LB-R and SsATF1-RB-F/SsATF1-RB-R, respectively. Two truncated and partially overlapped fragments of *HYG^R^* gene were separately PCR amplified from pDAN plasmid with the primers pDAN-F/LB-226-R and pDAN-R/RB-225-F. These PCR products served as templates in fusion PCR to generate two PCR fragments with the primers SsSLN1-LB-F/LB-226-R, RB-225-F/SsSLN1-RB-R, SsATF1-LB-F/LB-226-R and RB-225-F/SsATF1-RB-R, individually. The mixture of two fusion homologous fragments for each targeted gene was transformed into *MAT-1* and *MAT-2* protoplasts via PEG-mediated protoplast transformation. The transformants were recovered in a regeneration medium impregnated with 200 mg/mL hygromycin B (Merck, Saint Louis, MO, USA). Putative deletion mutants were screened and identified by PCR amplification using the following primers: SsSLN1-inside-F/SsSLN1-inside-R, SsSLN1-outside-F/SsSLN1-outside-R, SsATF1-inside-F/SsATF1-inside-R and SsATF1-outside-F/SsATF1-outside-R. This operation was used to generate the double-deletion mutants *ss1sln1*Δ*gpa3*Δ, *ss1sln1*Δ*uac1*Δ and *ss1sln1*Δ*adr1*Δ, with the Zeocin resistance (*ZEO^R^*) cassette as selection marker in the *ss1sln1*Δ background. 

*SsSLN1* and *SsATF1* reintegration was carried out, as previously described [38]. The *HYG^R^* gene in the mutant was replaced with the reintegrated gene, together with the *ZEO^R^* gene as a selection marker, by a split marker approach. A fragment containing the native promoter and *SsSLN1* or *SsATF1* gene was PCR amplified with wild-type genomic DNA as a template, using the primers SsSLN1-COM-F/SsSLN1-COM-R and SsATF1-COM-F/SsATF1-COM-R, respectively. These PCR products were ligated with vector pEASY-COM containing *HYG^R^*-LB (*HYG^R^*-left homologous arm), *ZEO^R^* gene and *HYG^R^*-RB (*HYG^R^*-right homologous arm). Two reintegrated homologous fragments, one fragment including *HYG^R^*-LB, a complete target gene and partially overlapped fragments of the *ZEO^R^* gene, and the other containing partially overlapped fragments of the *ZEO^R^* gene and *HYG^R^*-RB, were PCR amplified with pEASY-*SsSLN1*-COM or pEASY-*SsATF1*-COM as a template using the primers COM-LB-F/COM-LB-R and COM-RB-F/COM-RB-R, respectively. Two reintegrated homologous fragments were individually transformed into *ss1sln1*Δ, *ss2sln1*Δ, *ss1atf1*Δ and *ss2atf1*Δ protoplasts through PEG-mediated protoplast transformation. Putative complementation transformants were selected with 100 mg/mL zeocin and identified with the primers SsSLN1-inside-F/SsSLN1-inside-R and SsATF1-inside-F/SsATF1-inside-R.

The IP locus, a DNA sequence without a function in *S. scitamineum* genomic DNA, was used as the target sequence for the overexpression of *SsPTP1*, *SsPTP2* and *SsPRF1*. It was replaced with the overexpressed gene together with the *ZEO^R^* gene as a selection marker by a split marker approach. The CD fragments of *SsPTP1*, *SsPTP2* and *SsPRF1* genes were PCR amplified with *S. scitamineum* cDNA as a template, using the primers SsPTP1-OE-F/SsPTP1-OE-R, SsPTP2-OE-F/SsPTP2-OE-R and SsPRF1-OE-F/SsPRF1-OE-R, individually. These PCR products were individually ligated with vector pEASY-OE containing IP-LB (IP-left homologous arm), a constitutive GPA promoter, *ZEO^R^* gene and IP-RB (IP-right homologous arm). Two overexpressed homologous fragments, one fragment including IP-LB, the GPA promoter fused with the target gene and partially overlapped fragments of the *ZEO^R^* gene, and the other containing partially overlapped fragments of the *ZEO^R^* gene and IP-RB, were PCR amplified with templates pEASY-*SsPTP1*-OE, pEASY-*SsPTP2*-OE or pEASY-*SsPRF1*-OE using the primers OE-LB-F/OE-LB-R and OE-RB-F/OE-RB-R, respectively. Two overexpressed homologous fragments were mixed and transformed into *MAT-1*, *MAT-2* or *ss1hog1*Δ protoplasts by PEG-mediated protoplast transformation, respectively. Putative overexpression transformants were selected with 100 mg/mL zeocin and identified with RT-qPCR using the primers qRT-SsPTP1-F/qRT-SsPTP1-R, qRT-SsPTP2-F/qRT-SsPTP2-R and qRT-SsPRF1-F/qRT-SsPRF1-R. 

*Ss1Hog1:eGFP*(*sln1*Δ), *Ss1Hog1:RFP*, *Ss1Atf1:eGFP* and *Ss1Atf1:eGFP*(*hog1*Δ) strains were generated by using the termination codon of *SsHOG1* or *SsATF1* as the target sequence, which was replaced with a DNA sequence containing eGFP/RFP and *ZEO^R^* genes by a split marker approach. The left and/or right borders of *SsHOG1:eGFP*, *SsHOG1:RFP* and *SsATF1:eGFP* were separately PCR amplified from wild-type *MAT-1* genomic DNA, using the primers SsHog1:eGFP-LB-F/SsHog1:eGFP-LB-R, SsHog1:eGFP-RB-F/SsHog1: eGFP-RB-R, SsHog1:RFP-LB-F/SsHog1:RFP-LB-R, SsHog1:RFP-RB-F/SsHog1: RFP-RB-R, SsAtf1:eGFP-LB-F/SsAtf1:eGFP-LB-R and SsAtf1:eGFP-RB-F/SsAtf1: eGFP-RB-R. Two truncated and partially overlapped fragments of the *ZEO^R^* or *HYG^R^* gene, including *eGFP* or *RFP*, were PCR amplified from pEASY-*eGFP* or pEASY-*RFP* plasmid with the primers eGFP-LB-F/eGFP-LB-R, eGFP-RB-F/eGFP-RB-R, RFP-LB-F/RFP-LB-R and RFP-RB-F/RFP-RB-R, respectively. These PCR products served as templates in fusion PCR to generate two PCR fragments with the primers SsHog1:eGFP-LB-F/eGFP-LB-R, eGFP-RB-F/SsHog1:eGFP-RB-R, SsAtf1:eGFP-LB-F/eGFP-LB-R, eGFP-RB-F/SsAtf1:eGFP-RB-R and SsHog1:RFP-LB-F/RFP-LB-R, RFP-RB-F/SsHog1: RFP-RB-R, individually.

The mixture of two fusion homologous fragments was transformed into *MAT-1* or *ss1sln1*Δ protoplasts via PEG-mediated protoplast transformation for each targeted gene. The transformants were recovered in a regeneration medium impregnated with 200 mg/mL hygromycin B or 100 mg/mL zeocin. Putative fluorescent strains were screened and identified by fluorescence microscope and PCR amplification, respectively. The primers and sequences used in this study are listed in Table S1. Details of the strains generated and used in this study are listed in Table 1.

### 2.3. Nucleic Acid Related Manipulation

The *S. scitamineum* strains were grown on YePSA medium for 1–2 days at 28 °C. Then, the strains were collected and rapidly ground in liquid nitrogen to extract the genomic DNA of *S. scitamineum* using a modified SDS-based method [2]. The fresh haploid sporidia grown on YePSA medium at 28 °C for 30 h were used for the total RNA extraction of *S. scitamineum* with TRIzol reagent (ThermoFisher Scientific, Carlsbad, CA, USA) following established protocol [2]. HiScript ^®^ II 1st Strand cDNA Synthesis Kit (Vazyme, Nanjing, China) was used to synthesize the cDNA. Real-Time Quantitative PCR was performed using Fast SYBR™ Green Master Mix (ThermoFisher Scientific, Carlsbad, CA, USA) on QuantStudio 6 Flex (ThermoFisher Scientific, Carlsbad, CA, USA). The relative gene expression level was calculated by adopting the −ΔΔCt method [43] and the cytoskeletal protein gene *ACTIN* was used as an internal control [38]. The experiment was carried out in triplicate for three independent biological replicates. To carry out Southern blot analysis, genomic DNA of negative control *MAT-1* and *MAT-2*, positive control pDAN plasmid and genomic DNA of deletion mutant were digested with the restriction enzyme *Hind* III. The HPT sequence was amplified as the probe with PCR DIG Labeling Mix (Roche, Mannheim, BW, Germany). Probe hybridization was performed with a DIG Easy Hyb (Roche, Mannheim, BW, Germany) and detected by CSPD (Roche, Mannheim, BW, Germany). Probed bands of >3.0 kb size in deletion mutants confirmed the correct gene replacement. 

### 2.4. Sporidia Staining and Epifluorescence Microscopy

The haploid sporidia of *Ss1Hog1:RFP*, *Ss1Hog1:eGFP*(*sln1*Δ), *Ss1Atf1:eGFP* and *Ss1Atf1:eGFP*(*hog1*Δ) strains were cultured overnight in YePS liquid medium at 28 °C, diluted to O.D.600 = 0.2 with the fresh YePS liquid medium and grown up to O.D.600 = 1.0. The fresh haploid sporidia were collected for nucleic acid staining by centrifugation and washed twice with 1 × PBS. Then, the cells were re-suspended in 20 μL of the Antifade Mounting Medium with DAPI (Beyotime, Shanghai, China). Finally, the samples (10 μL) were mounted on the slide and photographed under a Leica DMI8 Inverted Fluorescence Microscope using DAPI, GFP and RFP filters. The pictures were taken through Leica Application Suite (LAS) v.X software. Scale bar = 10 μm.

### 2.5. SsHog1 Phosphorylation Assays

The total protein was extracted from the fresh haploid sporidia grown on YePSA medium at 28 °C for 30 h [38]. Protein samples were separated by 10% SDS-PAGE. Phosphorylation of SsHog1 was determined by Western blot analysis with an antibody Phospho-p38 MAPK (Thr180/Tyr182) (D3F9) XP ^®^ Rabbit (Cell Signaling Technology, Boston, MA, USA). Total levels of SsHog1 were detected by probing with an anti-Hog1 antibody (Genecreate Biological Engineering Company, Wuhan, China). Blot signals were displayed using enhanced chemiluminescence (BIO-RAD, 170-5061) after the binding of an Anti-Rabbit IgG–Peroxidase secondary antibody (Sigma, Louis, MO, USA), as described previously [38].

### 2.6. Yeast Two-Hybrid (Y2H) Assays

A modified yeast two-hybrid (Y2H) assay was used in this experiment [29]. The cDNA of *SsSLN1*, *SsADR1*, *SsUBC1* and *SsPRF1* was PCR amplified with Phanta ^®^ Max Super-Fidelity DNA Polymerase (Vazyme, Nanjing, China). The PCR products were individually cloned into pGBKT7 or pGADT7 vectors. After the sequence verification, the pGBKT7-SsSln1, pGADT7-SsAdr1, pGADT7-SsUbc1 and pGADT7-SsPrf1 plasmids were, respectively, introduced into the yeast *Y2HGold* strain. Finally, the transformants grown on a synthetic medium lacking tryptophan and leucine (SD-Trp-Leu) were transferred to a synthetic medium lacking tryptophan, leucine, histidine and adenine (SD-Trp-Leu-His-Ade) at 30 °C.

### 2.7. Assessment of Pathogenicity and Relative Fungal Biomass

A highly susceptible sugarcane cultivar ROC22 was used for the pathogenicity assay as described [2,39]. The haploid sporidia of *S. scitamineum* strains were grown on YePS medium in a shaking incubator at 28 °C for 1–2 days. The fresh haploid sporidia were collected, re-suspended in the sterilized ddH_2_O, adjusted to 1 × 10^6^ cells/mL and mixed with haploid sporidia of opposite mating types in equal volume. Sugarcane seedlings of ROC22 grown to 5–6 leaf stage were inoculated by injection with approximately 0.2 mL of the mixture per plant. *MAT-1* and *MAT-2* mixture served as a positive control. Inoculated plants were kept in the greenhouse and a natural cycle of day and night was followed for 3–6 months. Three biological repeats were performed in the inoculation and each replicate involved the infection of at least 15 plants. The symptoms of ‘black whip’ were documented and photographed at about six months post inoculation. Percentage (%) of ‘black whip’/total seedlings was estimated. 

Fungal biomass assay of the inoculated sugarcane seedlings was carried out according to Sun [44]. The same amount of *S. scitamineum* sporidia (mixture of compatible mating types) was injected into sugarcane seedlings at 3 days post-inoculation (dpi), and total DNA of the inoculated sugarcane tissue was extracted. The relative fungal biomass was measured using the fungal *ACTIN* gene as a reference, whereas the sugarcane glyceraldehyde dehydrogenase (*GAPDH*) gene served as an internal control.

### 2.8. Statistical Analysis

Data were expressed as mean ± standard error (SE). Differences among different treatments were analyzed using GraphPad Prism v.5 software.

## 3. Results

### 3.1. Identification of SsSln1 Protein in S. scitamineum

**Figure 1:**
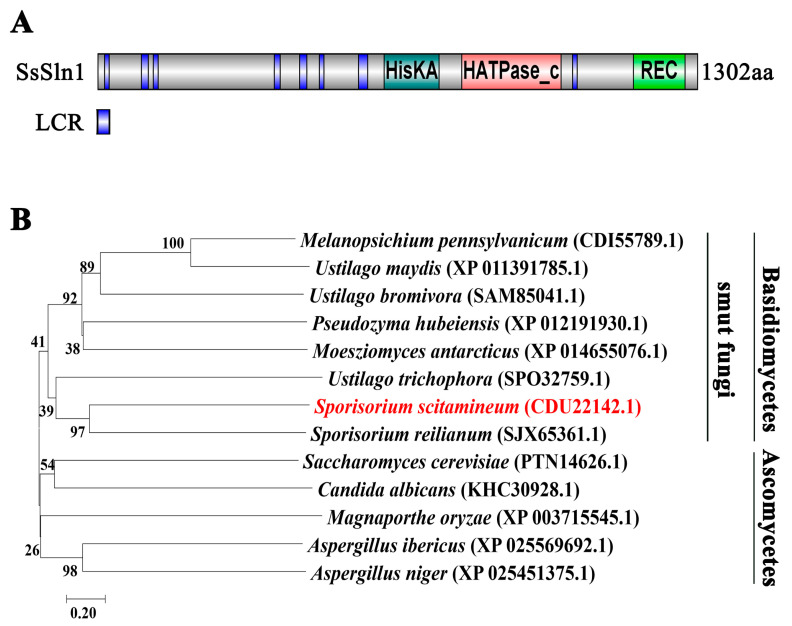
Phylogenetic analysis of SsSln1 protein. (**A**) Domain arrangement of *S. scitamineum* SsSln1 protein was performed using the SmartBLAST tool (https://blast.ncbi.nlm.nih.gov/smartblast/ (accessed on 8 June 2021)). LCR represents low-complexity regions. (**B**) The amino acid sequence of SsSln1 protein was used to search orthologous proteins through the BLASTp tool. The phylogenetic tree was constructed from the Sln1 proteins of *Melanopsichium pennsylvanicum* (CDI55789.1), *U. maydis* (XP 011391785.1), *Ustilago bromivora* (SAM85041.1), *Pseudozyma hubeiensis* (XP 012191930.1), *Moesziomyces antarcticus* (XP 014655076.1), *U. trichophora* (SPO32759.1), *S. scitamineum* (CDU22142.1), *S. reilianum* (SJX65361.1), *S. cerevisiae* (PTN14626.1), *C. albicans* (KHC30928.1), *M. oryzae* (XP 003715545.1), *Aspergillus ibericus* (XP 025569692.1) and *Aspergillus niger* (XP 025451375.1) using neighbor-joining method in MEGA 7.0 software [45]. Gap extension = 0.2.

A BLASTp search of *Sporisorium reilianum* Sln1 (SJX65361.1) protein sequence revealed that the *S. scitamineum* proteome harbors a putative histidine kinase osmosensor protein (CDU22142.1), which we named SsSln1. SsSln1 was predicted as a peptide of 1302 amino acids. Putative domains of SsSln1 protein were further predicted through the SmartBLAST tool (https://blast.ncbi.nlm.nih.gov/smartblast/ (accessed on 8 June 2021)), which revealed that SsSln1 had a conserved histidine kinases (HisKA) domain, a histidine kinase-like ATPases (HATPase_c) domain and a cheY-homologous receiver (REC) domain (Figure 1A). Moreover, the SsSln1 protein and its orthologs from other fungal species, including basidiomycetous and ascomycetous, were selected for phylogenetic analysis. The results indicate that SsSln1 was closely related to its orthologs in *S. reilianum* and *Ustilago trichophora*, the smut fungi phylogenetic clade, whereas it was distant to *Saccharomyces cerevisiae* (TPN14626.1) and *C. albicans* (KHC30928.1), which were present in another clade of the phylogenetic tree (Figure 1B).

Overall, we identified the putative histidine kinase SsSln1 in *S. scitamineum*, and found that SsSln1 was highly conserved with its orthologs in basidiomycetous fungi.

### 3.2. Deletion of SsSLN1 Gene Promotes Mating and Pathogenicity of S. scitamineum

To characterize the role of SsSln1 in *S. scitamineum*, the deletion mutants of *ss1sln1*Δ in *MAT-1* and *ss2sln1*Δ in *MAT-2* background were separately generated, whereas reintegrated mutants of *Ss1SLN1-COM* in *ss1sln1*Δ and *Ss2SLN1-COM* in *ss2sln1*Δ background were generated by following the homologous recombination approach, as described before [38,41]. Mutants were confirmed by PCR amplification (Figure S1A,B) and Southern blotting (Figure S2A). Real-Time Quantitative PCR (RT-qPCR) analysis confirmed the complete deletion of *SsSLN1* gene in *ss1sln1*Δ and *ss2sln1*Δ, and verified that *SsSLN1* gene fully recovered in *Ss1SLN1-COM* and *Ss2SLN1-COM* strains, respectively (Figure S2B). *S. scitamineum* wild-type (WT) strains and generated mutants generated and used in this study are listed in Table 1.

**Figure 2:**
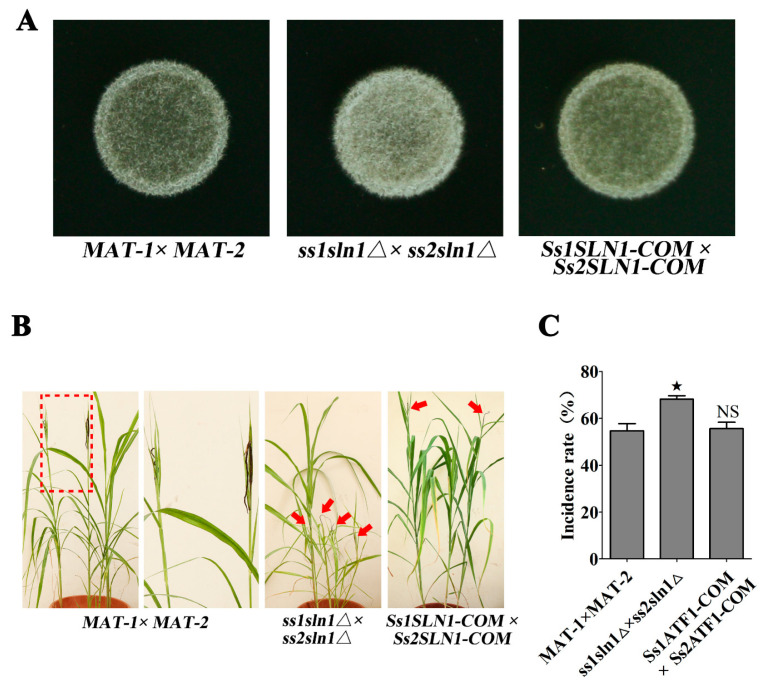
Effect of SsSln1 on mating and virulence. (**A**) Mating assay of mutants. Fresh haploid sporidia were grown up to O.D.600 = 1.0, and then mixed with an equal volume of the compatible WT strain or mutants and spotted onto YePSA medium to incubate at 28 °C. Images were taken after 30 h of cultivation. (**B**) Pathogenicity assay of *SsSln1* null mutants. A susceptible sugarcane variety ROC22 was inoculated with mixed sporidia (*1*:*1*) of *S. scitamineum* and wild-type or mutant combination via injection at the 5–6-leaf seedling stage. The infection assays were performed with at least 15 seedlings. The symptoms of ‘black whip’ were documented and photographed at about six months post inoculation. The red dotted box regions were enlarged for a better view of whip symptoms. The symptoms of ‘black whip’ are denoted by red arrows. (**C**) Bar chart depicts the quantification of infection as shown in (**B**). Percentage (%) of ‘black whip’/total seedlings is indicated. Bar chart depicts the statistical differences among the mean values (^★^ *p* < 0.05). Mean ± S.E. were derived from two independent biological repeats with three replications.

To evaluate the impact of SsSln1 on mating, the haploid cells of *MAT-1*, *MAT-2*, *ss1sln1*Δ, *ss2sln1*Δ, *Ss1SLN1-COM* and *Ss2SLN1-COM* were mixed with the sporidia of opposite mating type and inoculated on YePSA medium. Successful formation of dikaryotic hyphae was observed as the appearance of white, fuzzy colonies at 30 h post inoculation. However, the *ss1sln1*Δ × *ss2sln1*Δ combination exhibited a stronger white and fuzzy colony as compared to *MAT-1* × *MAT-2* (Figure 2A). Meanwhile, the reintegrated strain of the *Ss1SLN1-COM* × *Ss2SLN1-COM* combination displayed a semblable mating of *MAT-1* × *MAT-2* on the same plate (Figure 2A). To examine the role of SsSln1 in virulence, the sporidial suspensions of *MAT-1* × *MAT-2*, *ss1sln1*Δ × *ss2sln1*Δ and *Ss1SLN1-COM* × *Ss2SLN1-COM* combination were inoculated on the susceptible sugarcane cultivar ROC22. The results show that the typical symptoms of ‘black whip’ disease were observed in all strain-infected seedlings. However, the disease symptoms of *ss1sln1*Δ × *ss2sln1*Δ were more conspicuous than the wild-type or reintegrated strains (Figure 2B). Statistical results reveal that 68.15% of the total seedlings infected with *ss1sln1*Δ × *ss2sln1*Δ displayed ‘black whip’ symptoms, which was significantly higher (*p* < 0.05) than the incidence in other in treatments (Figure 2C).

In short, the SsSln1 negatively regulated the mating and virulence of *S. scitamineum*.

### 3.3. SsSln1 Negatively Regulates Phosphorylation and Nuclear Localization of SsHog1

**Figure 3:**
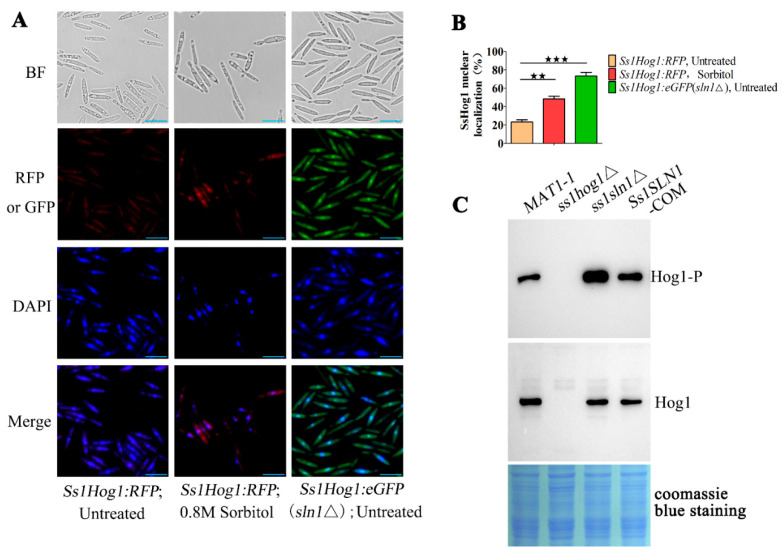
Effect of SsSln1 on phosphorylation and subcellular localization of SsHog1. (**A**) Subcellular localization of SsHog1 protein in *S. scitamineum*. The *Ss1Hog1:RFP* and *Ss1Hog1:eGFP*(*sln1*Δ) sporidia were grown up to O.D.600 = 1.0, and then treated with or without 0.8 M sorbitol in YePSA medium at 28 °C for 60 min. The nucleus was stained with DAPI and the images were taken under fluorescence microscope. Scale bar = 10 μm. (**B**) Bar chart depicts the quantification of nuclear localization as shown in (**A**). Percentage (%) of nuclear localization cells/total cells is indicated. Bar chart depicts the statistical difference among the mean values (^★★^ *p* < 0.01, ^★★★^ *p* < 0.001). Mean ± S.E. were derived from three independent biological repeats with three replications. (**C**) Phosphorylation of SsHog1 was enhanced in *ss1sln1*Δ mutant cells. The fresh haploid sporidia were grown in YePSA medium at 28 °C for 30 h. The total protein of sporidia was extracted with lysis buffer. Western blots depicting the levels of SsHog1 phosphorylation in the indicated strains. Above: blots were probed for phosphorylated SsHog1 (Hog1-P). Below: blots were probed for total SsHog1 (Hog1). Coomassie blue staining of total proteins served as loading control.

The Sln1–Ypd1–Ssk1 “two-component” system regulates the Hog1–MAP kinase cascade in the budding yeast [7]. To explore the effects of SsSln1 on SsHog1 activity, we constructed a *Ss1Hog1:RFP* fusion strain in *MAT-1* and *Ss1Hog1:eGFP*(*sln1*Δ) fusion strain in *ss1sln1*Δ. The localization analysis was carried out by staining the cell of *Ss1Hog1:RFP* and *Ss1Hog1:eGFP*(*sln1*Δ) fusion strains with 4′,6-diamidino-2-phenylindole (DAPI), photographed under a fluorescence microscope. In the absence of osmotic stress conditions, the SsHog1:RFP fusion protein was mainly distributed in the cytoplasm (Figure 3A), whereas the SsHog1 protein signal accumulated in the nucleus of about 23.3% of cells (Figure 3B). However, 0.8 M sorbitol treatment enhanced the entry of SsHog1:RFP fusion protein signals, and the number of cells increased up to 48.4% of cells (Figure 3A,B). Surprisingly, in *ss1sln1*Δ mutants, a large amount of SsHog1:eGFP fusion protein concentrated in the nucleus (Figure 3A), and the proportion of SsHog1 protein nuclear localization was noted in about 73.3% of cells (Figure 3B). These results suggest that SsHog1 was activated in the absence of *SsSLN1* even without hyperosmosis treatment. To investigate the effect of SsSln1 on the phosphorylation level of SsHog1 in *S. scitamineum*, the level of SsHog1 phosphorylation was examined in *MAT-1*, *ss1hog1*Δ, *ss1sln1*Δ and *Ss1SLN1-COM* sporidia by Western blotting. The SsHog1 was found to be highly activated and phosphorylated in *ss1sln1*Δ mutants as compared to wild-type and *Ss1SLN1-COM*, and the phosphorylation of SsHog1 was abolished in *ss1hog1*Δ (Figure 3C).

Taken together, the SsSln1 negatively regulated the phosphorylation and nuclear localization of SsHog1 in *S. scitamineum*.

### 3.4. Phosphorylation of SsHog1 Is Necessary for Mating and Virulence of S. scitamineum

**Figure 4:**
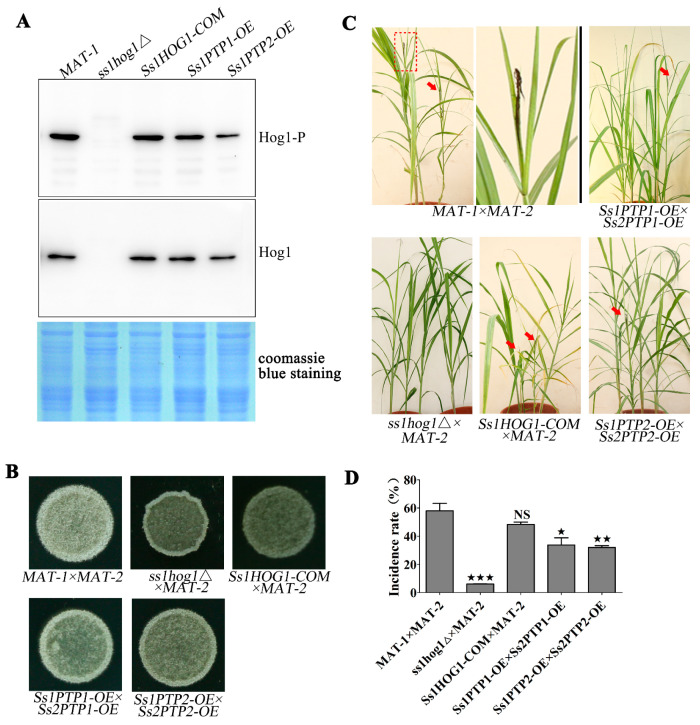
The effect of reduced SsHog1 phosphorylation level on mating and virulence. (**A**) Detection of SsHog1 phosphorylation in *S. scitamineum*. The fresh haploid sporidia were grown in YePSA medium at 28 °C for 30 h. The total protein of *S. scitamineum* was extracted with lysis buffer. Western blots depicting the levels of SsHog1 phosphorylation in the indicated strains. Above: blots were probed for phosphorylated SsHog1 (Hog1-P). Below: blots were probed for total SsHog1 (Hog1). Coomassie blue staining of total proteins served as loading control. (**B**) Mating assay of mutants. The fresh haploid sporidia were grown up to O.D.600 = 1.0, and then mixed with an equal volume of WT or mutant sporidia of opposite mating types and spotted onto YePSA medium to incubate at 28 °C. Images were taken after 30 h of cultivation. (**C**) Pathogenicity assay of mutants. *MAT-1* × *MAT-2*, *Ss1PTP1-OE* × *Ss2PTP1-OE*, *Ss1PTP2-OE* × *Ss2PTP2-OE*, *Ss1HOG1-COM* × *MAT-2* and *ss1hog1*Δ × *MAT-2* combination were inoculated into the sugarcane seedlings of variety ROC22. The infection assays were performed with at least 15 seedlings. The symptoms of ‘black whip’ were documented and photographed at about six months post inoculation. The symptoms of ‘black whip’ are denoted by red arrows. The red dotted box regions were enlarged for a better view of whip symptoms. (**D**) Bar chart depicts the quantification of infection as shown in (**C**). Percentage (%) of ‘black whip’/total seedlings was indicated. Bar chart depicts the statistical difference among the mean values (^★^ *p* < 0.05, ^★★^ *p* < 0.01, ^★★★^ *p* < 0.001). Mean ± S.E. were derived from two independent biological repeats with three replications.

The role of protein tyrosine phosphatase Ptps in the dephosphorylation of Hog1 orthologs in *C. neoformans* was reported [30]. Therefore, we first identified the putative Ptps orthologs SsPtp1 (CDU22358.1) or SsPtp2 (CDU23562.1), and generated the overexpression mutants of *SsPTP1-OE* or *SsPTP2-OE* in *MAT-1* and *MAT-2* background, respectively (Figures S1E and S2D,E). As expected, the overexpression of *Ss1PTP2-OE* exhibited a lower level of SsHog1 phosphorylation (Figure 4A) in comparison to the wild type. To investigate the effects of the SsHog1 phosphorylation level on the mating and virulence of *S. scitamineum*, mating assays were performed by cospotting the compatible strains onto YePSA medium. The results reveal that during mating, the white hyphae was slightly reduced in the *Ss1PTP1-OE* × *Ss2PTP1-OE* combination but significantly reduced in the *Ss1PTP2-OE* × *Ss2PTP2-OE* combination, compared to the wild type (Figure 4B). In addition, the *ss1hog1*Δ mutant was also noted to be significantly deficient in mating in comparison to wild type and *Ss1HOG1-COM* (Figure 4B). Further pathogenicity analysis revealed that the typical symptoms of the disease ‘black whip’ were significantly (*p* < 0.05) reduced in *ss1hog1*Δ × *MAT-2* combination, as compared to the wild-type and reintegrated strains (Figure 4C). Moreover, the percentage (%) of ‘black whip’/total seedlings was also markedly reduced in *ss1hog1*Δ × *MAT-2* combination than wild-type or *Ss1HOG1-COM* × *MAT-2* combination (Figure 4D). The inoculation of *Ss1PTP1-OE* × *Ss2PTP1-OE* and *Ss1PTP2-O*E × *Ss2PTP2-OE* combination also produced the ‘black whip’ symptoms but their virulence was significantly more reduced than the wild-type (Figure 4D).

Overall, the results demonstrate that SsPtp2 was involved in the dephosphorylation of SsHog1 and resulted in defective mating and virulence in *S. scitamineum*.

### 3.5. Phosphorylation Level of SsHog1 Positively Mediates Mating and Virulence of S. scitamineum

An Atf1 ortholog named SsAtf1 was identified and characterized in *S. scitamineum*. Deletion mutants of *ss1atf1*Δ in *MAT-1* and *ss2atf1*Δ in *MAT-2*, and reintegrated mutants of *Ss1ATF1-COM* in *ss1atf1*Δ and *Ss2ATF1-COM* in *ss2atf1*Δ background, were individually obtained by homologous recombination approach as described before [38]. Mutants were confirmed by PCR amplification (Figure S1A,B) and Southern blot (Figure S2A). The results of RT-qPCR analysis revealed the complete deletion of the *SsATF1* gene in *ss1atf1*Δ and *ss2atf1*Δ, and it was fully recovered in the reintegrated strains *Ss1ATF1-COM* and *Ss2ATF11-COM*, respectively (Figure S2C). 

**Figure 5:**
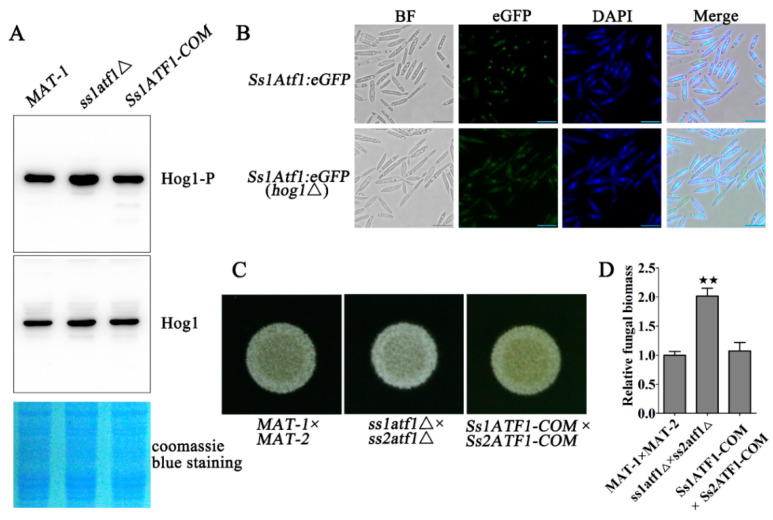
Effect of SsAtf1 on SsHog1 phosphorylation and *S. scitamineum* mating. (**A**) Phosphorylation of SsHog1 was increased in *ss1atf1*Δ mutant cells. The fresh haploid sporidia were grown in YePSA medium at 28 °C for 30 h. The total protein of *S. scitamineum* was extracted with lysis buffer. Western blots depicting the levels of SsHog1 phosphorylation in the indicated strains. Above: blots were probed for phosphorylated SsHog1 (Hog1-P). Below: blots were probed for total SsHog1 (Hog1). Coomassie blue staining of total proteins served as loading control. (**B**) Subcellular localization of SsAtf1 protein in *S. scitamineum*. *Ss1Atf1:eGFP* and *Ss1Atf1:eGFP*(*hog1*Δ) sporidia were grown up to O.D.600 = 1.0, and stained with DAPI. Images were taken under the fluorescence microscope. Scale bar = 10 μm. (**C**) Mating assay of mutants. The fresh haploid sporidia were grown up to O.D.600 = 1.0, and then mixed with an equal volume of the compatible WT strain or mutants and spotted onto YePSA medium to incubate at 28 °C. Images were taken after 30 h of cultivation. (**D**) Measurement of relative fungal biomass. The fresh haploid sporidia were grown up to O.D.600 = 1.0, and then mixed with an equal volume of the compatible WT strain or mutants. The *MAT-1* × *MAT-2*, *ss1atf1*Δ × *ss2atf1*Δ, and *Ss1ATF1-COM* × *Ss2ATF1-COM* combination was equally inoculated into the sugarcane seedlings of variety ROC22 to incubate at 28 °C for 3 days. Relative fungal biomass was measured by RT-qPCR with the total DNA isolated from infected sugarcane stems. The fungal *ACTIN* gene was used for the estimation of relative fungal biomass through the −ΔΔCt method with the plant *GADPH* gene as an internal control. Bar chart depicts the statistical difference among the mean values (^★★^ *p* < 0.01). Mean ± S.E. were derived from two independent biological repeats with three replications.

To characterize the potential influence of SsAtf1 on the phosphorylation level of SsHog1, the levels of SsHog1 phosphorylation in *MAT-1*, *ss1atf1*Δ and *Ss1ATF1-COM* sporidia were assessed. As expected, the SsHog1 was activated and phosphorylated in *ss1atf1*Δ mutants as compared to wild-type and *Ss1ATF1-COM* (Figure 5A). The phosphorylation of Atf1 by *M. oryzae* Hog1 ortholog has been reported [29]. Therefore, a *Ss1Atf1:eGFP* fusion strain was generated in *MAT-1* or *ss1hog1*Δ to test its role in SsAtf1 nuclear localization. To study the localization, the *Ss1Atf1:eGFP* and *Ss1Atf1:eGFP*(*hog1*Δ) sporidia were stained with DAPI and observed under a fluorescence microscope. The results indicate that SsAtf1:eGFP fusion protein was mainly accumulated in the nucleus of both *MAT-1* and *ss1hog1*Δ strains (Figure 5B), suggesting that SsHog1 did not affect the nuclear localization of SsAtf1. Furthermore, the mating with wild-type, deletion of *SsATF1* and reintegrated strains was also assessed. The results depict an increase in the formation of white and fuzzy colonies in *ss1atf1*Δ × *ss2atf1*Δ combination in comparison to wild-type and reintegrated strains (Figure 5C). The relative fungal biomass continuously and significantly increased for up to 3 days in seedling stems inoculated with *ss1atf1*Δ × *ss2atf1*Δ, as compared to that *MAT-1* × *MAT-2* or *Ss1ATF1-COM* × *Ss2ATF1-COM* (Figure 5D).

These data demonstrate that SsAtf1 contributed to the dephosphorylation of SsHog1, enhanced SsHog1 phosphorylation level and facilitated the mating or virulence of *S. scitamineum*.

### 3.6. SsSln1 Negatively Regulates the Transcription of SsPRF1 by SsHog1 Phosphorylation, but Not through the cAMP/PKA Signaling Pathway

**Figure 6:**
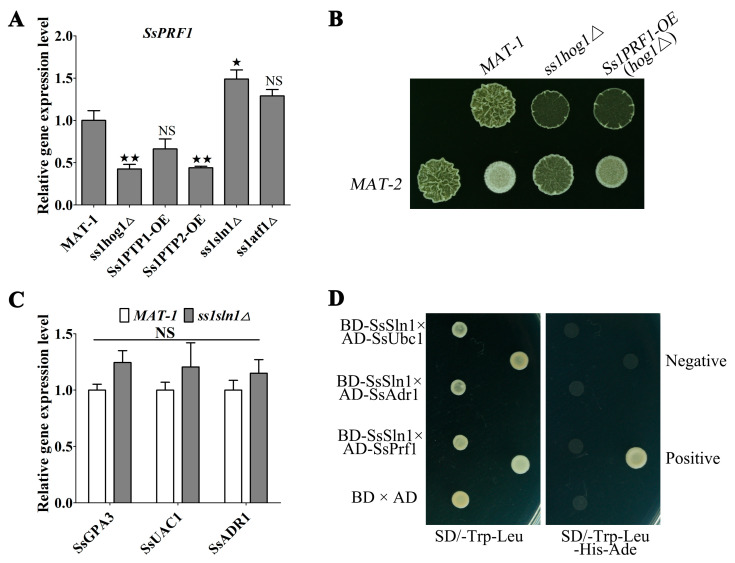
The relationship between SsSln1 and cAMP/PKA pathway in regulating the transcription of *SsPRF1*. (**A**) RT-qPCR analysis of *SsPRF1* gene expression in the *MAT-1*, *ss1hog1*Δ, *Ss1PTP1-OE*, *Ss1PTP2-OE*, *ss1sln1*Δ and *ss1atf1*Δ strains under sporidial growth on YePSA plate for 30 h, respectively. The relative gene expression level was calculated by following the −ΔΔCt method with *ACTIN* as an internal control. Bar chart depicts the statistical difference among the mean values (^★^ *p* < 0.05, ^★★^ *p* < 0.01). Mean ± S.E. were derived from three independent biological repeats with three replications. (**B**) Mating assay of mutants. Sporidia from *MAT-1*, *ss1hog1*Δ and *SsPRF1-OE*(*hog1*Δ) strains were separately mixed with an equal volume of *MAT-2* sporidia and spotted to YePSA medium to incubate at 28 °C. Images were taken after 40 h of cultivation. (**C**) Transcriptional profile of *SsGPA3*, *SsUAC1* and *SsADR1* gene in the *MAT-1* and *ss1sln1*Δ mutant. The relative gene expression level was calculated according to the −ΔΔCt method with *ACTIN* as an internal control. The NS represents no significance. Mean ± S.E. were derived from three independent biological repeats with three replications. (**D**) The yeast two-hybrid assay. Simultaneous co-transformation of positive-control vectors (pGBKT7-p53 and pGADT7-LargeT), negative-control vectors (pGADT7-LargeT and pGBKT7-LaminC), empty vectors (pGADT7 and pGBKT7) and pGBKT7-SsSln1 (bait vectors, BD) with pGADT7-SsAdr1, pGADT7-SsUbc1 and pGADT7-SsPrf1 into the Y2H Gold strain. The transformant was grown on SD-Trp-Leu (lacking tryptophan and leucine) and SD-Trp-Leu-His-Ade (lacking tryptophan, leucine, histidine and adenine) synthetic medium to incubate at 30 °C. Images were taken after 3 days of cultivation. Images are representative of *n* = 2 biological replications of the experiment.

Previously, our study showed that the pheromone response factor *SsPRF1* gene plays a key role in mating and pathogenicity [39], and was significantly down-regulated in cAMP/PKA defective mutants [38]. Therefore, RT-qPCR analysis was performed for assessing the expression of the *SsPRF1* gene in *MAT-1*, *ss1hog1*Δ, *Ss1PTP1-OE*, *Ss1PTP2-OE*, *ss1sln1*Δ and *ss1atf1*Δ strains grown on YePSA medium. The results show that transcriptional expression of the *SsPRF1* gene was significantly (*p* < 0.05) reduced in *ss1hog1*Δ and *Ss1PTP2-OE*, whereas it slightly decreased in *Ss1PTP1-OE* mutant, compared to wild type (Figure 6A). However, the transcription level of the *SsPRF1* gene was significantly (*p* < 0.05) up-regulated in *ss1sln1*Δ mutant, whereas it slightly increased in *ss1atf1*Δ mutant (Figure 6A). To evaluate whether the reduced mating in *ss1hog1*Δ mutants was caused by the down-regulation of the *SsPRF1* gene, a *Ss1PRF1-OE*(*hog1*Δ) mutant was generated, which overexpressed the *SsPRF1* gene in *ss1hog1*Δ mutants. The mating was noted to be fully restored in *Ss1PRF1-OE*(*hog1*Δ) mutant compared to the *ss1hog1*Δ mutant (Figure 6B), and the transcriptional expression of *SsPRF1* gene in *Ss1PRF1-OE*(*hog1*Δ) mutant was close to the level of wild-type *MAT-1* (Figure S2F). Furthermore, the transcriptional profiling showed that *SsGPA3*, *SsUAC1* and *SsADR1* genes were not significantly different in wild-type and *ss1sln1*Δ strains grown in YePSA medium (Figure 6C). Moreover, SsSln1 did not interact with the regulatory subunit SsUbc1 or the catalytic subunit SsAdr1 of cAMP-dependent protein kinase A (PKA) through yeast two-hybrid analysis, indicating that SsSln1 also did not interact with the pheromone response factor SsPrf1 in yeast two-hybrid analysis (Figure 6D).

These results collectively suggest that SsSln1 negatively regulates the transcription of *SsPRF1* by SsHog1 phosphorylation, but not through the cAMP/PKA signaling pathway.

### 3.7. Cross-Talk between the SsSln1 and cAMP/PKA Pathways Antagonistically Regulates Mating and Virulence by SsPRF1 Transcription

**Figure 7:**
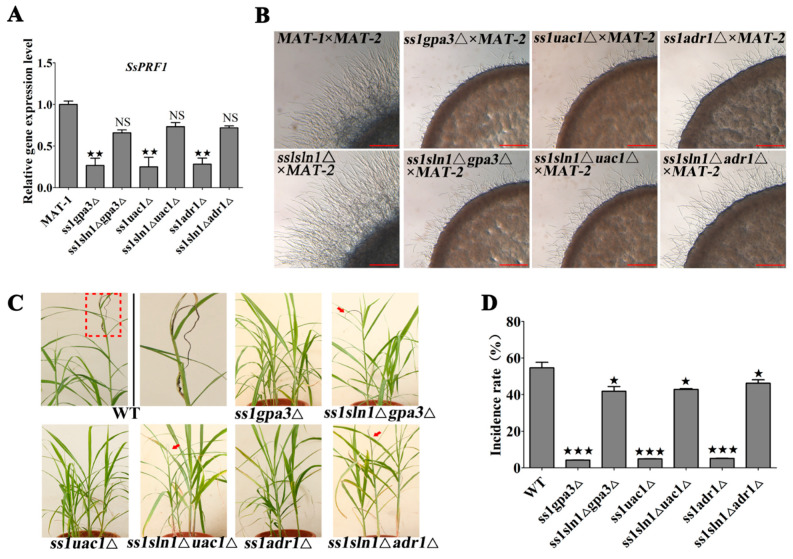
Effect of cross-talk between SsSln1 and cAMP/PKA pathway on mating and virulence. (**A**) Transcriptional profile of *SsPRF1* gene in the *MAT-1* and mutants. The RT-qPCR assay was performed to assess the expression of *SsPRF1* gene in *MAT-1*, *ss1gpa3*Δ, *ss1sln1*Δ*gpa3*Δ, *ss1uac1*Δ, *ss1sln1*Δ*uac1*Δ, *ss1adr1*Δ, and *ss1sln1*Δ*adr1*Δ strains under sporidial growth on YePSA plate for 30 h, respectively. The relative gene expression was calculated by following the −ΔΔCt method with *ACTIN* as an internal control. Bar chart depicts the statistical difference among the mean values (^★★^ *p* < 0.01). The NS represents no significance. Mean ± S.E. were derived from three independent biological repeats with three replications. (**B**) Mating assay of mutants. Sporidia from MAT, *ss1sln1*Δ, *ss1gpa3*Δ, *ss1sln1*Δ*gpa3*Δ, *ss1uac1*Δ, *ss1sln1*Δ*uac1*Δ, *ss1adr1*Δ and *ss1sln1*Δ*adr1*Δ strains were separately mixed with *MAT-2* sporidia of equal volume and spotted onto minimal medium to incubate at 28 °C. Images were taken after 30 h of cultivation. Scale bar = 0.5 mm. (**C**) Pathogenicity assay of mutants. Sporidia from *MAT-1*, *ss1gpa3*Δ, *ss1sln1*Δ*gpa3*Δ, *ss1uac1*Δ, *ss1sln1*Δ*uac1*Δ, *ss1adr1*Δ and *ss1sln1*Δ*adr1*Δ strains were separately mixed with an equal volume of *MAT-2* sporidia and inoculated into the sugarcane seedlings of variety ROC22. The infection assays were performed with at least 15 seedlings. The symptoms of ‘black whip’ were documented and photographed at about six months post inoculation. The red dotted box regions were enlarged for a better view of whip symptoms. The symptoms of ‘black whip’ are denoted by red arrows. (**D**) Bar chart depicts the quantification of infection as shown in (**C**). Percentage (%) of ‘black whip’/total seedlings is indicated. Bar chart depicts the statistical difference among the mean values (^★^ *p* < 0.05, ^★★★^ *p* < 0.001). Mean ± S.E. were derived from two independent biological repeats with three replications.

To investigate the potential relationship of SsSln1 with the cAMP/PKA pathway during mating and virulence of *S. scitamineum*, *SsGPA3*, *SsUAC1* and *SsADR1* deletion strains were individually generated in *ss1sln1*Δ mutants by homologous recombination and named as *ss1sln1*Δ*gpa3*Δ, *ss1sln1*Δ*uac1*Δ and *ss1sln1*Δ*adr1*Δ (Figures S1C,D and S2G,H). Transcriptional profiling revealed that the expression of pheromone response factor gene *SsPRF1* was significantly reduced in the cAMP/PKA defective mutants (Figure 7A), which is consistent with previous findings [38]. However, *SsPRF1* gene expression was not significantly reduced in *ss1sln1*Δ*gpa3*Δ, *ss1sln1*Δ*uac1*Δ and *ss1sln1*Δ*adr1*Δ mutants, as compared to the wild type (Figure 7A). To test the changes in the mating of double-deletion strains, we assessed the mating of *MAT-1*, *ss1sln1*Δ and cAMP/PKA single-deletion mutants (*ss1gpa3*Δ, *ss1uac1*Δ and *ss1adr1*Δ) and double-deletion strains (*ss1sln1*Δ*gpa3*Δ, *ss1sln1*Δ*uac1*Δ and *ss1sln1*Δ*adr1*Δ) after mixing with *MAT-2* sporidia on minimal medium. The results display that the mating capability of *ss1sln1*Δ*gpa3*Δ, *ss1sln1*Δ*uac1*Δ and *ss1sln1*Δ*adr1*Δ mutants could be partially restored in the *ss1gpa3*Δ, *ss1uac1*Δ and *ss1adr*1Δ mutants by mixing with compatible wild-type *MAT-2* (Figure 7B). 

Pathogenicity analysis was also performed for assessing the virulence in wild-type, cAMP/PKA-defective mutants, and double-deletion strains. The results show that the cAMP/PKA-defective mutants failed to induce the disease symptoms in sugarcane seedlings, as previously described [38]. Contrarily, typical disease ‘black whip’ symptoms were observed in the double-deletion strains and WT-infected seedlings (Figure 7C). Statistical analysis depicted that the double-deletion strains presented the disease symptoms in more than 40% of the seedlings, whereas 55% of wild-type seedlings exhibited disease symptoms (Figure 7D).

Taken together, these data suggest that cross-talk between histidine kinase SsSln1 and cAMP/PKA pathways regulates the mating and virulence by affecting the transcription of the *SsPRF1* gene.

## 4. Discussion

The two-component histidine kinases of fungi function as sensor proteins to mediate signal transduction events related to morphogenesis, cell growth, mycelium development, cell wall regulation, osmotic adaptation and virulence [7,15,46,47]. Ten histidine kinases have been reported in *M. oryzae* [6], one (Sln1) in *S. cerevisiae* [3,48] and three (Sln1, Hk1 and Nik1) in *C. albicans* [3,14]. During this study, a histidine kinase Sln1 was identified and functionally characterized in *S. scitamineum*. However, SLN1 is not essential for the growth of *S. scitamineum*, which contradicts the previous reports of *S. cerevisiae* [7,8,49].

**Figure 8:**
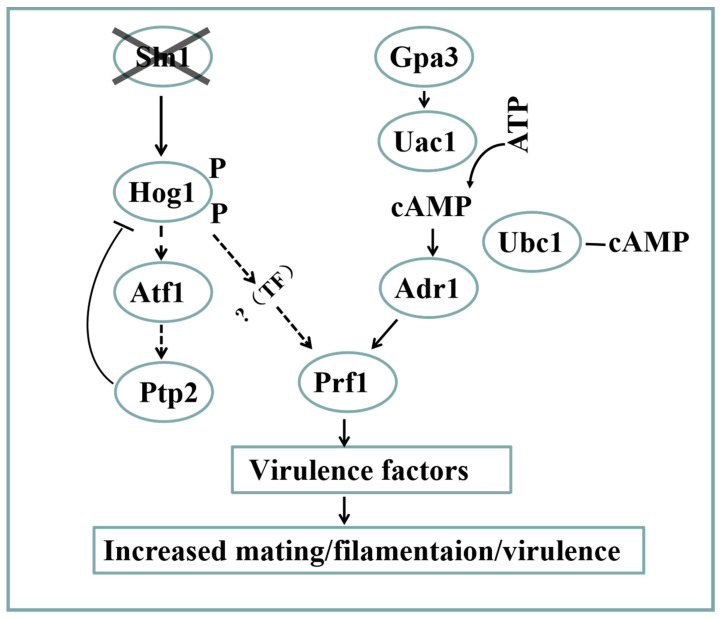
A proposed model depicting the outcomes after Sln1 loss in *S. scitamineum*. Loss of Sln1 results in increased phosphorylation of Hog1. The levels of Hog1 phosphorylation are suppressed via induction of the negative regulator Ptp2, which is initiated by the transcription factor Atf1. Loss of Sln1 function causes increased mating and virulence of *S. scitamineum*, by possibly enhancing Hog1 activity to promote the transcription of Prf1. Furthermore, the cAMP/PKA signaling pathway positively regulates mating and virulence by the transcription factor Prf1. TF represents a transcription factor.

Interestingly, our results show that the *SsSln1* null mutant exhibited a stronger mating activity and increased virulence in *S. scitamineum*, which is opposite to what has been reported in *C. albicans* [14]. One question raised by this study is why enhanced mating and virulence occur in the *SsSln1* null mutant. The model for regulating the mating and virulence after the loss of SsSln1 is summarized in Figure 8. In this study, an increased level of SsHog1 phosphorylation in the *ss1sln1*Δ mutant was found, indicating that SsSln1 negatively regulates SsHog1 phosphorylation in *S. scitamineum*. This is in line with previous reports about *S. cerevisiae* [7,48], which revealed the activation of Hog1 kinase through disruption of the *SLN1* gene. However, *ss1sln1*Δ mutant displayed enhanced SsHog1 phosphorylation, mating and virulence that was similar to the *Ypd1* null mutant of *C. albicans*, which blocked the *YPD1* gene to enhance Hog1 phosphorylation and virulence [13]. This suggests that SsSln1-mediated SsHog1 might regulate the mating and virulence of *S. scitamineum*. The Hog1 kinase was reported to regulate virulence in various pathogenic fungi, such as *C. albicans* [49], *C. neoformans* [50,51] and *Aspergillus fumigatus* [9,52,53], but not in *M. oryzae* [54]. This study also elaborated that the elimination or reduction of SsHog1 phosphorylation affected the mating and virulence of *S. scitamineum*. Atf1 ortholog was also identified and characterized during the study, which was reported in *M. oryzae* [29,55] and *C. neoformans* [30] to suppress the hyperphosphorylation of Hog1 ortholog. As expected, the deletion of *SsATF1* enhanced the phosphorylation of SsHog1, as compared to the wild-type and *Ss1ATF1-COM* strains. In *M. oryzae*, the Atf1 is required for virulence [29,55]. We found that *SsAtf1* null mutant enhanced the mating and increased relative fungal biomass in inoculated seedlings in comparison to wild type. Nevertheless, the potential role of SsAtf1 in enhancing *S. scitamineum* virulence should be further investigated before assessing the pathogenicity with inoculation. In short, the results of this study demonstrate that the phosphorylation of SsHog1 was required for the mating and virulence of *S. scitamineum*.

On the other hand, several signal transduction pathways may interact with each other in fungi [37]. A significant down-regulation of pheromone response factor *SsPRF1* gene in cAMP/PKA-defective mutants and *sskpp2*Δ mutant was reported in our previous study [38,56]. Moreover, the exogenous addition of cAMP could partially restore the mating defect in *sskpp2*Δ mutant [56], suggesting that there are essential connections between the cAMP/PKA and Kpp2 MAPK pathways in *S. scitamineum*. In this study, our findings reveal an intriguing underlying relationship between the histidine kinase SsSln1 and cAMP/PKA pathways during mating and virulence. We found that the histidine kinase SsSln1 did not interact with the regulatory subunit SsUbc1, catalytic subunit SsAdr1 of cAMP-dependent protein kinase A (PKA) and pheromone response factor SsPrf1 by yeast two-hybrid analysis. This is inconsistent with the results reported in *M. oryzae*, where interaction between Sln1 and the regulatory subunit of cAMP-dependent protein kinase A (Sum1) was demonstrated by yeast two-hybrid analyses and co-immunoprecipitation [15]. In addition, the abolition of SsHog1 phosphorylation led to the down-regulated transcription of the *SsPRF1* gene. Contrarily, the enhanced SsHog1 phosphorylation up-regulated *SsPRF1* transcription, suggesting that histidine kinase SsSln1 negatively regulated SsHog1 phosphorylation to activate the transcription of the *SsPRF1* gene. However, the mechanism of SsHog1 phosphorylation that regulates the transcription of the *SsPRF1* gene requires further elaboration. The pheromone response factor SsPrf1 was reported to play a key role in the virulence factor of *S. scitamineum* [39]. During this study, we found that the mating could be fully restored in *Ss1PRF1-OE*(*hog1*Δ) mutant, implying that the down-regulation of the *SsPRF1* gene decreased the mating ability of *ss1hog1*Δ mutant. The transcription of the *SsPRF1* gene was also partly restored in the double-deletion strains (*ss1sln1*Δ*gpa3*Δ, *ss1sln1*Δ*uac1*Δ and *ss1sln1*Δ*adr1*Δ), as compared to down-regulation in cAMP/PKA-defective mutants, as previously described [38]. Meanwhile, the double-deletion strains exhibited remarkably enhanced mating and virulence capabilities in comparison to the cAMP/PKA-defective mutants. Taken together, we infer that cross-talk between the SsSln1 and cAMP/PKA pathways antagonistically regulates mating and virulence by balancing the transcription of the *SsPRF1* gene in *S. scitamineum*. 

In short, this study provides evidence that histidine kinase SsSln1 negatively regulates SsHog1 phosphorylation, which is essential for mating and virulence in *S. scitamineum*. Furthermore, we also reveal a novel mechanism by which histidine kinase SsSln1 and cAMP/PKA pathways antagonistically regulate mating and virulence via affecting the transcription of the *SsPRF1* gene. 

## Figures and Tables

**Table 1:** Details of strains generated in this study.

Strains	Accession Number for Protein	Resistance Marker and Strain Background	Reference or Source
*MAT-1*		*a1*, *b1*	[2]
*MAT-2*		*a2*, *b2*	[2]
*ss1sln1*Δ	CDU22142.1	Hygromycin, *MAT-1*	This study
*ss2sln1*Δ	CDU22142.1	Hygromycin, *MAT-2*	This study
*Ss1SLN1-COM*	CDU22142.1	Zeocin, *ss1sln1*Δ	This study
*Ss2SLN1-COM*	CDU22142.1	Zeocin, *ss2sln1*Δ	This study
*ss1atf1*Δ	CDU21933.1	Hygromycin, *MAT-1*	This study
*ss2atf1*Δ	CDU21933.1	Hygromycin, *MAT-2*	This study
*Ss1ATF1-COM*	CDU21933.1	Zeocin, *ss1atf1*Δ	This study
*Ss2ATF1-COM*	CDU21933.1	Zeocin, *ss2atf1*Δ	This study
*ss1hog1*Δ	CDU23149.1	Hygromycin, *MAT-1*	[41]
*Ss1PTP1-OE*	CDU22358.1	Zeocin, *MAT-1*	This study
*Ss2PTP1-OE*	CDU22358.1	Zeocin, *MAT-2*	This study
*Ss1PTP2-OE*	CDU23562.1	Zeocin, *MAT-1*	This study
*Ss2PTP2-OE*	CDU23562.1	Zeocin, *MAT-2*	This study
*Ss1PRF1-OE*(*hog1*Δ)	CDU22680.1	Zeocin, *ss1hog1*Δ	This study
*Ss1Hog1:RFP*	CDU23149.1	Hygromycin, *MAT-1*	This study
*Ss1Hog1:eGFP*(*sln1*Δ)	CDU23149.1	Zeocin, *ss1sln1*Δ	This study
*Ss1Atf1:eGFP*	CDU21933.1	Zeocin, *MAT-1*	This study
*Ss1Atf1:eGFP*(*hog1*Δ)	CDU21933.1	Zeocin, *ss1hog1*Δ	This study
*ss1sln1*Δ*gpa3*Δ	CDU22378.1	Zeocin, *ss1sln1*Δ	This study
*ss1sln1*Δ*uac1*Δ	CDU25762.1	Zeocin, *ss1sln1*Δ	This study
*ss1sln1*Δ*adr1*Δ	CDU22361.1	Zeocin, *ss1sln1*Δ	This study

## Data Availability

All data required to understand this article are presented in the study or the Supplementary Materials. Any raw data further requested will be provided by the corresponding authors.

## Supplementary Materials

The following are available online at https://www.mdpi.com/article/10.3390/jof7080610/s1, Figure S1: Identification of mutants by PCR amplification, Figure S2: Southern blot and RT-qPCR analysis of the mutant, Table S1: The primers and sequences used in this study.

## References

[1] Taniguti L.M., Schaker P.D., Benevenuto J., Peters L.P., Carvalho G., Palhares A., Quecine M.C., Nunes F.R., Kmit M.C., Wai A. (2015). Complete Genome Sequence of *Sporisorium scitamineum* and Biotrophic Interaction Transcriptome with Sugarcane. PLoS ONE.

[2] Yan M., Zhu G., Lin S., Xian X., Chang C., Xi P., Shen W., Huang W., Cai E., Jiang Z. (2016). The mating-type locus b of the sugarcane smut *Sporisorium scitamineum* is essential for mating, filamentous growth and pathogenicity. Fungal Genet. Biol..

[3] Saito H. (2001). Histidine phosphorylation and two-component signaling in eukaryotic cells. Chem. Rev..

[4] Catlett N.L., Yoder O.C., Turgeon B.G. (2003). Whole-genome analysis of two-component signal transduction genes in fungal pathogens. Eukaryot. Cell.

[5] Motoyama T., Ochiai N., Morita M., Iida Y., Usami R., Kudo T. (2008). Involvement of putative response regulator genes of the rice blast fungus *Magnaporthe oryzae* in osmotic stress response, fungicide action, and pathogenicity. Curr. Genet..

[6] Zhang H., Liu K., Zhang X., Song W., Zhao Q., Dong Y., Guo M., Zheng X., Zhang Z. (2010). A two-component histidine kinase, MoSLN1, is required for cell wall integrity and pathogenicity of the rice blast fungus, *Magnaporthe oryzae*. Curr. Genet..

[7] Posas F., Wurgler-Murphy S.M., Maeda T., Witten E.A., Thai T.C., Saito H. (1996). Yeast HOG1 MAP kinase cascade is regulated by a multistep phosphorelay mechanism in the SLN1-YPD1-SSK1 “two-component” osmosensor. Cell.

[8] Hohmann S. (2002). Osmotic stress signaling and osmoadaptation in yeasts. Microbiol. Mol. Biol. Rev..

[9] Alex L.A., Borkovich K.A., Simon M.I. (1996). Hyphal development in *Neurospora crassa*: Involvement of a two-component histidine kinase. Proc. Natl. Acad. Sci. USA.

[10] Schumacher M.M., Enderlin C.S., Selitrennikoff C.P. (1997). The osmotic-1 locus of *Neurospora crassa* encodes a putative histidine kinase similar to osmosensors of bacteria and yeast. Curr. Microbiol..

[11] Furukawa K., Katsuno Y., Urao T., Yabe T., Yamada-Okabe T., Yamada-Okabe H., Yamagata Y., Abe K., Nakajima T. (2002). Isolation and functional analysis of a gene, tcsB, encoding a transmembrane hybrid-type histidine kinase from *Aspergillus nidulans*. Appl. Environ. Microbiol..

[12] Fassler J.S., West A.H. (2013). Histidine phosphotransfer proteins in fungal two-component signal transduction pathways. Eukaryot. Cell.

[13] Day A.M., Smith D.A., Ikeh M.A., Haider M., Herrero-de-Dios C.M., Brown A.J., Morgan B.A., Erwig L.P., MacCallum D.M., Quinn J. (2017). Blocking two-component signalling enhances *Candida albicans* virulence and reveals adaptive mechanisms that counteract sustained SAPK activation. PLoS Pathog..

[14] Yamada-Okabe T., Mio T., Ono N., Kashima Y., Matsui M., Arisawa M., Yamada-Okabe H. (1999). Roles of three histidine kinase genes in hyphal development and virulence of the pathogenic fungus *Candida albicans*. J. Bacteriol..

[15] Ryder L.S., Dagdas Y.F., Kershaw M.J., Venkataraman C., Madzvamuse A., Yan X., Cruz-Mireles N., Soanes D.M., Oses-Ruiz M., Styles V. (2019). A sensor kinase controls turgor-driven plant infection by the rice blast fungus. Nature.

[16] Posas F., Saito H. (1997). Osmotic activation of the HOG MAPK pathway via Ste11p MAPKKK: Scaffold role of Pbs2p MAPKK. Science.

[17] Posas F., Saito H. (1998). Activation of the yeast SSK2 MAP kinase kinase kinase by the SSK1 two-component response regulator. EMBO J..

[18] Tatebayashi K., Yamamoto K., Tanaka K., Tomida T., Maruoka T., Kasukawa E., Saito H. (2006). Adaptor functions of Cdc42, Ste50, and Sho1 in the yeast osmoregulatory HOG MAPK pathway. EMBO J..

[19] Maeda T., Takekawa M., Saito H. (1995). Activation of yeast PBS2 MAPKK by MAPKKKs or by binding of an SH3-containing osmosensor. Science.

[20] Van Wuytswinkel O., Reiser V., Siderius M., Kelders M.C., Ammerer G., Ruis H., Mager W.H. (2000). Response of *Saccharomyces cerevisiae* to severe osmotic stress: Evidence for a novel activation mechanism of the HOG MAP kinase pathway. Mol. Microbiol..

[21] O’Rourke S.M., Herskowitz I. (2004). Unique and redundant roles for HOG MAPK pathway components as revealed by whole-genome expression analysis. Mol. Biol. Cell.

[22] Horie T., Tatebayashi K., Yamada R., Saito H. (2008). Phosphorylated Ssk1 prevents unphosphorylated Ssk1 from activating the Ssk2 mitogen-activated protein kinase kinase kinase in the yeast high-osmolarity glycerol osmoregulatory pathway. Mol. Cell. Biol..

[23] Zarrinpar A., Bhattacharyya R.P., Nittler M.P., Lim W.A. (2004). Sho1 and Pbs2 act as coscaffolds linking components in the yeast high osmolarity MAP kinase pathway. Mol. Cell.

[24] Hohmann S. (2009). Control of high osmolarity signalling in the yeast *Saccharomyces cerevisiae*. FEBS Lett..

[25] Ferrigno P., Posas F., Koepp D., Saito H., Silver P.A. (1998). Regulated nucleo/cytoplasmic exchange of HOG1 MAPK requires the importin beta homologs NMD5 and XPO1. EMBO J..

[26] Reiser V., Ruis H., Ammerer G. (1999). Kinase activity-dependent nuclear export opposes stress-induced nuclear accumulation and retention of Hog1 mitogen-activated protein kinase in the budding yeast *Saccharomyces cerevisiae*. Mol. Biol. Cell.

[27] Sharma P., Mondal A.K. (2005). Evidence that C-terminal non-kinase domain of Pbs2p has a role in high osmolarity-induced nuclear localization of Hog1p. Biochem. Biophys. Res. Commun..

[28] Hersen P., McClean M.N., Mahadevan L., Ramanathan S. (2008). Signal processing by the HOG MAP kinase pathway. Proc. Natl. Acad. Sci. USA.

[29] Liu X., Zhou Q., Guo Z., Liu P., Shen L., Chai N., Qian B., Cai Y., Wang W., Yin Z. (2020). A self-balancing circuit centered on MoOsm1 kinase governs adaptive responses to host-derived ROS in *Magnaporthe oryzae*. eLife.

[30] Lee K.T., Byun H.J., Jung K.W., Hong J., Cheong E., Bahn Y.S. (2014). Distinct and redundant roles of protein tyrosine phosphatases Ptp1 and Ptp2 in governing the differentiation and pathogenicity of *Cryptococcus neoformans*. Eukaryot. Cell.

[31] Pan X., Harashima T., Heitman J. (2000). Signal transduction cascades regulating pseudohyphal differentiation of *Saccharomyces cerevisiae*. Curr. Opin. Microbiol..

[32] Thevelein J.M., Cauwenberg L., Colombo S., De Winde J.H., Donation M., Dumortier F., Kraakman L., Lemaire K., Ma P., Nauwelaers D. (2000). Nutrient-induced signal transduction through the protein kinase A pathway and its role in the control of metabolism, stress resistance, and growth in yeast. Enzym. Microb. Technol..

[33] Hogan D.A., Sundstrom P. (2009). The Ras/cAMP/PKA signaling pathway and virulence in *Candida albicans*. Future Microbiol..

[34] Choi J., Jung W.H., Kronstad J.W. (2015). The cAMP/protein kinase A signaling pathway in pathogenic basidiomycete fungi: Connections with iron homeostasis. J. Microbiol..

[35] Marroquin-Guzman M., Wilson R.A. (2015). GATA-Dependent Glutaminolysis Drives Appressorium Formation in *Magnaporthe oryzae* by Suppressing TOR Inhibition of cAMP/PKA Signaling. PLoS Pathog..

[36] Caza M., Kronstad J.W. (2019). The cAMP/Protein Kinase a Pathway Regulates Virulence and Adaptation to Host Conditions in *Cryptococcus neoformans*. Front. Cell. Infect. Microbiol..

[37] Martínez-Soto D., Ortiz-Castellanos L., Robledo-Briones M., León-Ramírez C.G. (2020). Molecular Mechanisms Involved in the Multicellular Growth of Ustilaginomycetes. Microorganisms.

[38] Chang C., Cai E., Deng Y.Z., Mei D., Qiu S., Chen B., Zhang L.H., Jiang Z. (2019). cAMP/PKA signalling pathway regulates redox homeostasis essential for *Sporisorium scitamineum* mating/filamentation and virulence. Environ. Microbiol..

[39] Zhu G., Deng Y., Cai E., Yan M., Cui G., Wang Z., Zou C., Zhang B., Xi P., Chang C. (2019). Identification and Functional Analysis of the Pheromone Response Factor Gene of *Sporisorium scitamineum*. Front. Microbiol..

[40] Garrido E., Pérez-Martín J. (2003). The crk1 gene encodes an Ime2-related protein that is required for morphogenesis in the plant pathogen *Ustilago maydis*. Mol. Microbiol..

[41] Cai E., Li L., Deng Y., Sun S., Jia H., Wu R., Zhang L., Jiang Z., Chang C. (2021). MAP kinase Hog1 mediates a cytochrome P450 oxidoreductase to promote the *Sporisorium scitamineum* cell survival under oxidative stress. Environ. Microbiol..

[42] Cai E., Mei D., Zhang X., Sun X., Li L., Wu R., Deng Y., Jiang Z., Chang C. (2020). A gene knockout method based on protoplast transformation with two PCR fragments in *Sporisorium scitamineum*. Mycosystema.

[43] Livak K.J., Schmittgen T.D. (2001). Analysis of relative gene expression data using real-time quantitative PCR and the 2(-Delta Delta C(T)) Method. Methods.

[44] Sun S., Deng Y., Cai E., Yan M., Li L., Chen B., Chang C., Jiang Z. (2019). The Farnesyltransferase β-Subunit Ram1 Regulates *Sporisorium scitamineum* Mating, Pathogenicity and Cell Wall Integrity. Front. Microbiol..

[45] Kumar S., Stecher G., Tamura K. (2016). MEGA7: Molecular Evolutionary Genetics Analysis Version 7.0 for Bigger Datasets. Mol. Biol. Evol..

[46] Calderone R.A., Fonzi W.A. (2001). Virulence factors of *Candida albicans*. Trends Microbiol..

[47] Lipa P., Janczarek M. (2020). Phosphorylation systems in symbiotic nitrogen-fixing bacteria and their role in bacterial adaptation to various environmental stresses. PeerJ.

[48] Maeda T., Wurgler-Murphy S.M., Saito H. (1994). A two-component system that regulates an osmosensing MAP kinase cascade in yeast. Nature.

[49] Alonso-Monge R., Navarro-García F., Molero G., Diez-Orejas R., Gustin M., Pla J., Sánchez M., Nombela C. (1999). Role of the mitogen-activated protein kinase Hog1p in morphogenesis and virulence of *Candida albicans*. J. Bacteriol..

[50] Bahn Y.S., Kojima K., Cox G.M., Heitman J. (2005). Specialization of the HOG pathway and its impact on differentiation and virulence of *Cryptococcus neoformans*. Mol. Biol. Cell.

[51] Ko Y.J., Yu Y.M., Kim G.B., Lee G.W., Maeng P.J., Kim S., Floyd A., Heitman J., Bahn Y.S. (2009). Remodeling of global transcription patterns of *Cryptococcus neoformans* genes mediated by the stress-activated HOG signaling pathways. Eukaryot. Cell.

[52] Winkelströter L.K., Bom V.L., de Castro P.A., Ramalho L.N., Goldman M.H., Brown N.A., Rajendran R., Ramage G., Bovier E., Dos Reis T.F. (2015). High osmolarity glycerol response PtcB phosphatase is important for *Aspergillus fumigatus* virulence. Mol. Microbiol..

[53] Bruder Nascimento A.C., Dos Reis T.F., de Castro P.A., Hori J.I., Bom V.L., de Assis L.J., Ramalho L.N., Rocha M.C., Malavazi I., Brown N.A. (2016). Mitogen activated protein kinases SakA(HOG1) and MpkC collaborate for *Aspergillus fumigatus* virulence. Mol. Microbiol..

[54] Dixon K.P., Xu J.R., Smirnoff N., Talbot N.J. (1999). Independent signaling pathways regulate cellular turgor during hyperosmotic stress and appressorium-mediated plant infection by *Magnaporthe grisea*. Plant Cell.

[55] Guo M., Guo W., Chen Y., Dong S., Zhang X., Zhang H., Song W., Wang W., Wang Q., Lv R. (2010). The basic leucine zipper transcription factor Moatf1 mediates oxidative stress responses and is necessary for full virulence of the rice blast fungus *Magnaporthe oryzae*. Mol. Plant Microbe Interact..

[56] Deng Y.Z., Zhang B., Chang C., Wang Y., Lu S., Sun S., Zhang X., Chen B., Jiang Z. (2018). The MAP Kinase SsKpp2 Is Required for Mating/Filamentation in *Sporisorium scitamineum*. Front. Microbiol..

