# Supplementary material for: Histidine Kinase Sln1 and cAMP/PKA Signaling Pathways Antagonistically Regulate Sporisorium scitamineum Mating and Virulence via Transcription Factor Prf1
Source: J Fungi (Basel). 2021 Jul 28;7(8):610. doi: 10.3390/jof7080610 (PMC8397173; doi:10.3390/jof7080610)
Supplement: Supplementary file 1 [file jof-07-00610-s001.zip › jof-1291942-supplementary.pdf]

Journal of Fungi

Supplementary Material

Histidine Kinase Sln1 and cAMP/PKA Signaling Pathways Antagonistically Regulate *Sporisorium scitamineum* Mating and Virulence via Transcription Factor Prf1

Enping Cai [1,2,†], Shuquan Sun [1,3,†], Yizhen Deng [1,2], Peishen Huang [1,2], Xian Sun [1,2], Yuting Wang [2], Changqing Chang [1,2,*] and Zide Jiang [1,*]

[1] College of Plant Protection, South China Agricultural University, Guangzhou 510642, China; dlcep@foxmail.com (E.C.); sunshuquan@yrcti.edu.cn (S.S.); dengyz@scau.edu.cn (Y.D.); hps547384492@gmail.com (P.H.); sunx2021@163.com (X.S.)

[2] Integrate Microbiology Research Center, Guangdong Province Key Laboratory of Microbial Signals and Disease Control, South China Agricultural University, Guangzhou 510642, China; yutingwbio@163.com

[3] Environmental monitoring and Remediation Engineering Technology Research Center, School of Environmental Engineering, Yellow River Conservancy Technical Institute, Kaifeng 475004, China

* Correspondence: zdjiang@scau.edu.cn (Z.J.); changcq@scau.edu.cn (C.C.); Tel: +86-020-3860-4779 (Z.J.); Tel: +020-86-757-3225 (C.C.)

† Co-first authors

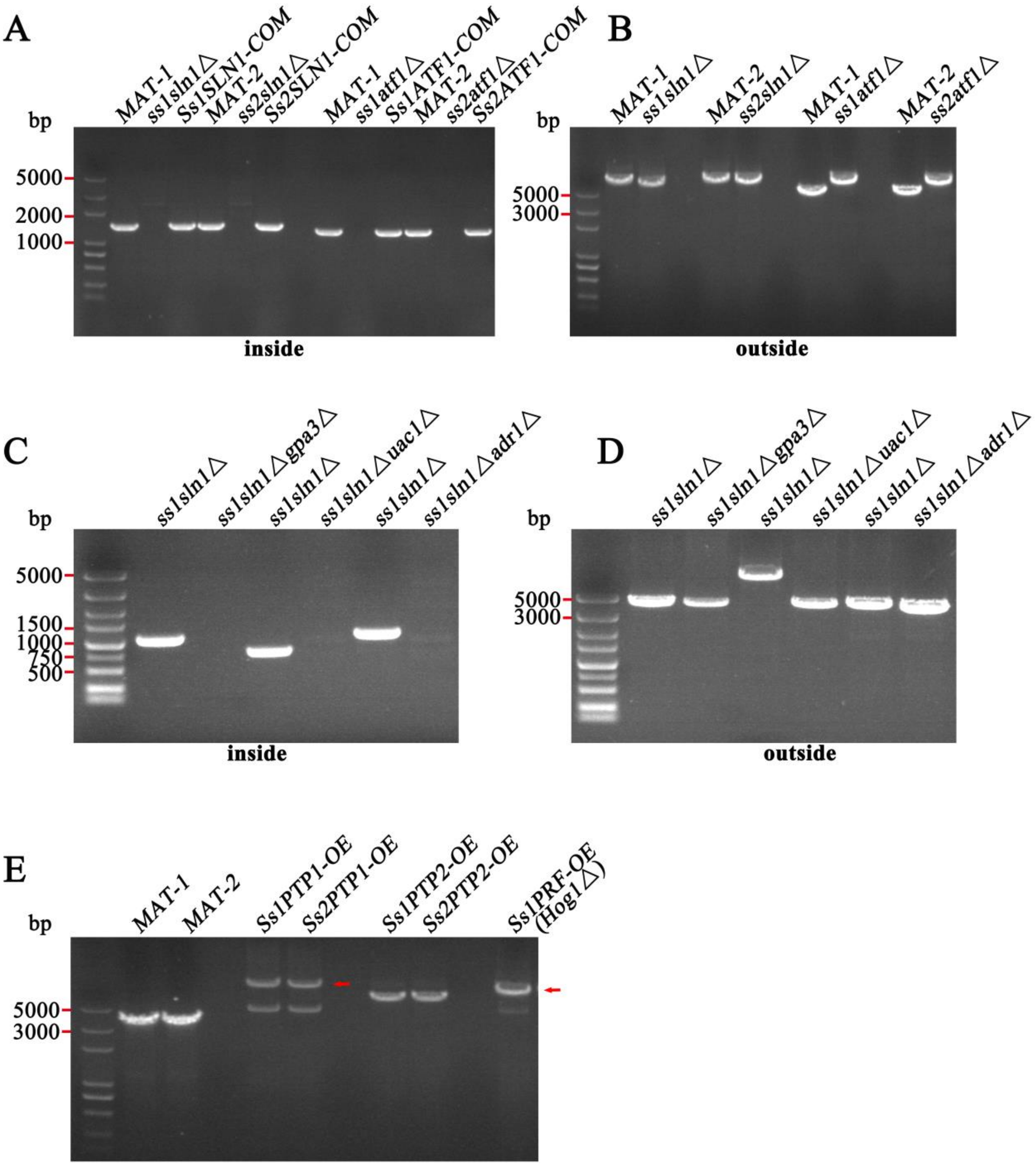

Figure 1. Identification of mutants by PCR amplification. (**A**) and (**B**) PCR amplification was performed with specific primers inside-F/inside-F and outside-F/outside-F (listed in Table S1) to confirm the replacement of targeted gene with the *HPT*^{R} selection marker (gene deletion) or replacement of *HPT*^{R} selection marker with targeted gene (gene complementation). Molecular markers in bp were labeled. (**C**) and (**D**) PCR amplification was performed with specific primers inside-F/inside-F and outside-F/outside-F (listed in Table S1) to confirm the replacement of targeted gene with the *ZEO*^{R} selection marker. Molecular markers in bp were labeled. (**E**) PCR amplification was performed with universal primers OE-JC-F/OE-JC-R (listed in Table S1) to confirm the replacement of targeted sequence with the *ZEO*^{R} selection marker and targeted gene. Molecular markers in bp were labeled. The target band was denoted with red arrows.

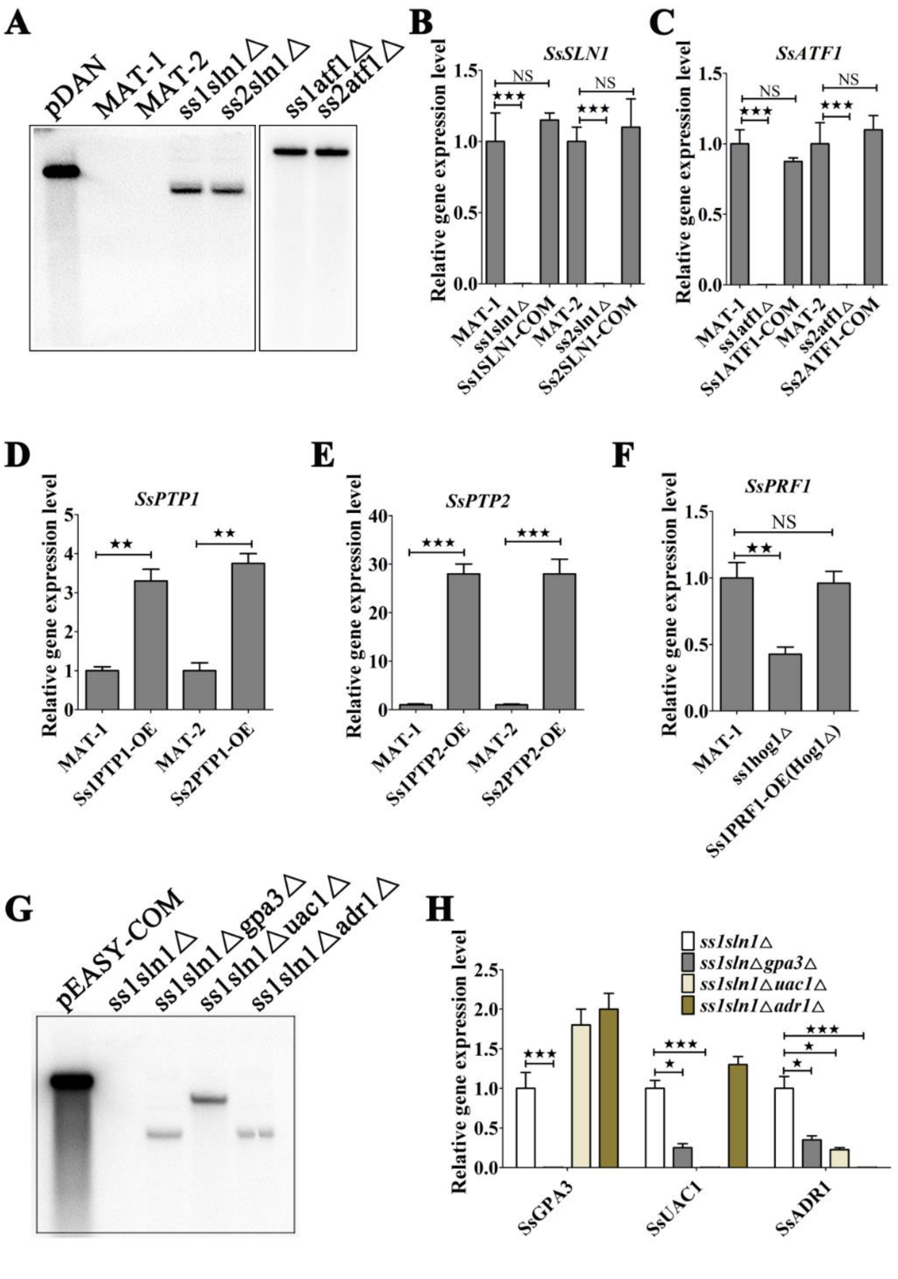

Figure 2. Southern blot and RT-qPCR analysis of the mutant. (**A**) and (**G**) Southern blot analysis was performed for confirming the deletion mutants. The genomic DNA of *MAT-1*, *MAT-2*, *ss1sln1*△, *ss1sln2*△, *ss1atf1*△, *ss1atf2*△, *ss1sln1*△*gpa3*△, *ss1sln1*△*uac1*△, *sss1sln1*△*adr1*△, and the pDAN and pEASY-COM plasmid were digested with the restriction enzyme *Hind*III at 37 °C for overnight. The *HPT* gene was used as the probe in (**A**). The 5755 bp band of the pDAN plasmid served as a positive control of the experimental procedure. Probed bands of 4661 bp and 12200 bp size in the *ss1sln1*△, *ss1sln2*△, *ss1atf1*△, and *ss1atf2*△ mutants confirmed the correct gene replacement events, respectively. The *ZEO* gene was used as the probe in (**G**). The 7056 bp band of the pEASY-COM plasmid served as a positive control of the experimental procedure. Probed bands of 2330 bp, 5132 bp and 2374 bp size in the *ss1sln1*△*gpa3*△, *ss1sln1*△*uac1*△, and *sss1sln1*△*adr1*△ mutants confirmed the correct gene replacement events, severally. (**B–F**) and (**H**) RT-qPCR analysis for expression of *SsSLN1*, *SsATF1*, *SsPTP1*, *SsPTP2*, *SsPRF1*, *SsGPA31*, *SsUAC1*, and *SsADR1* genes in the wild type and mutants under sporidial growth on YePSA plate for 24 h, respectively. The relative gene expression level was calculated by following the -△△Ct method with *ACTIN* as an

internal control. Barchart depicts statistical difference among the mean values (* $p < 0.05$, ** $p < 0.01$, *** $p < 0.001$). The NS represents no significance. Mean ± S.E. were derived from three independent biological repeats with three replications.

Table S1. The primers and sequences used in this study.

The primers	Sequence (5′- - 3′)
The primers for gene deletion	
pDAN-LB-F	CAGGGTTTTCCCAGTCACGAC
LB-226-R	GGTCAAGACCAATGCGGAGC
pDAN-RB-R	TCACACAGGAAACAGCTATGACC
RB-225-F	GCAAGACCTGCCTGAAACCG
SsSLN1-LB-F	TTGCTCTTCGGGACTTGTGGG
SsSLN1-LB-R	GTCGTGACTGGGAAAACCCTGTGAGGGCAAGCATGGGTGAA
SsSLN1-RB-F	GGTCATAGCTGTTTCCTGTGTGATGGCAAGGTGAATGGCGAGA
SsSLN1-RB-R	CGAACGAGCCAAGCAAGTCG
SsATF1-LB-F	GTGCGTGACGATGACGGAGA
SsATF1-LB-R	GTCGTGACTGGGAAAACCCTGGAACGAAACCGACAAGCGGC
SsATF1-RB-F	GGTCATAGCTGTTTCCTGTGTGAGCCGGATGATGGTGGTAGGG
SsATF1-RB-R	CTATTGATGCGCGCTGGTCG
ZEO-LB-F	GATAGTTTAAACTGAAGGCGGG
ZEO-LB-R	GAAGTGCACGCAGTTGCCG
ZEO-RB-F	CAAGAACAAGCGCTGTCGCC
ZEO-RB-R	AGCGGGCAGTTCGGTTTCA
SsGPA3-LB-F	GAGGCAAGCCAAGCCAGTTGA
SsGPA3-LB-R	CCCGCCTTCAGTTTAAACTATCTTTGGGTGTGTGTGAGAGGCT
SsGPA3-RB-F	TGAAACCGAACTGCCCGCTTCGGCCAAGAAGCAGCGAT
SsGPA3-RB-R	AGAAGCGATCAGCAAGCAAGCA
SsUAC1-LB-F	ATGCAATCTGCACTTCGGCC
SsUAC1-LB-R	CCCGCCTTCAGTTTAAACTATCCTTCTTTGGGTTGCTCTCGTGC
SsUAC1-RB-F	TGAAACCGAACTGCCCGCTATGGTGGCCAGATCCTGGCTA
SsUAC1-RB-R	AGGAAGCAGATCCACCAGTCG
SsADR1-LB-F	CTTGTTGAGAGGTGGGCGAT
SsADR1-LB-R	CCCGCCTTCAGTTTAAACTATCGAGTTGGCGTGATGAGACGG
SsADR1-RB-F	TGAAACCGAACTGCCCGCTAGAGGAGGATTTGCGAGCG
SsADR1-RB-R	GATGAGCGTGGATGCGTTTG
The primers for gene complementation	
SsSLN1-COM-F	ATCTGATCCAAGCTCAAGCTCGAAGCGAACCGTGGAAACG
SsSLN1-COM-R	AGCAAGATCTAATCAAGCTTAAAATCAAACGCCCCCACGC
SsATF1-COM-F	ATCTGATCCAAGCTCAAGCTGTAGAAGCCATAGCGTGCCG
SsATF1-COM-R	AGCAAGATCTAATCAAGCTTCAAAGGAGCGAGGGGATGGA
COM-LB-F	CCTCGAGCGATCCTTGAAGC
COM-LB-R	AGCGGGCAGTTCGGTTTCA
COM-RB-F	CAAGAACAAGCGCTGTCGCC
COM-RB-R	CGAGCATTCACTAGGCAACCA
The primers for gene overexpression	
SsPTP1-OE-F	AACCAAAACACTCTTCCACCATGTCGACGCAACCTCCAGA
SsPTP1-OE-R	AGCAAGATCTAATCAAGCTTTCACACAACCCCTTCGCGCTGCA
SsPTP2-OE-F	AACCAAAACACTCTTCCACCATGTCACTAGGCATCCGACC
SsPTP2-OE-R	AGCAAGATCTAATCAAGCTTCTACTTGGCCAGCCCAGCTTCCC
SsPRF1-OE-F	AACCAAAACACTCTTCCACCATGCGAGACCAAGCTACCACG
SsPRF1-OE-R	AGCAAGATCTAATCAAGCTTCTACGTCGAGGCGGACTGCTG

OE-LB-F	GAAAGGTGCGACGGTGTGC
OE-LB-R	GGCTGTCGAAAGGTCAGGTCT
OE-RB-F	AGTTGACCAGTGCCGTTCCG
OE-RB-R	AGCGACGAACCTTGCCATCA
OE-JC-F	CGGATGATCGCGGGATAGGG
OE-JC-R	GATCCGGTCTCTGTCCGCAG
The primers for the fluorescent protein fusion strain were constructed	
SsHog1:RFP-LB-F	CAGTCTGGCCATCGCTTCGT
SsHog1:RFP-LB-R	ATGACGTCCTCGGAGGAGGCGTTATTGACGGGCGGCACG
SsHog1:RFP-RB-F	GGTCATAGCTGTTTCCTGTGTGAACTACAGCGTCCGAGCCTTG
SsHog1: RFP-RB-R	GACGACCTGGTGCTTGACGA
RFP-LB-F	GCCTCCTCCGAGGACGTCAT
RFP-LB-R	AGCGGGCAGTTCGGTTTCA
RFP-RB-F	TATCCAGGCAACAGGGCACC
RFP-RB-R	TCACACAGGAAACAGCTATGACC
SsHog1:eGFP-LB-F	CAGTCTGGCCATCGCTTCGT
SsHog1:eGFP-LB-R	GCTCCTCGCCCTTGCTCACGTTATTGACGGGCGGCACG
SsHog1:eGFP-RB-F	GGCCGAGGAGCAGGACTGAACTACAGCGTCCGAGCCTTG
SsHog1: eGFP-RB-R	GACGACCTGGTGCTTGACGA
SsAtf1:eGFP-LB-F	GCCGCCATGGTCAACAACAC
SsAtf1:eGFP-LB-R	GCTCCTCGCCCTTGCTCACCACCTTGATGGGAGCGTCTG
SsAtf1:eGFP-RB-F	GGCCGAGGAGCAGGACTGACGCTCCTTTGTACCACGCTTTC
SsAtf1: eGFP-RB-R	ACGCGCTGTACTGGCTGAAT
eGFP-LB-F	GTGAGCAAGGGCGAGGAGC
eGFP-LB-R	CTCGCCGATCTCGGTCATGG
eGFP-RB-F	GTTGCCCGTGTTTCTCGCTG
eGFP-RB-R	TCAGTCCTGCTCCTCGGCC
The primers for gene detection	
SsSLN1-inside-F	TCCACTTCAGCAGTCGCTCG
SsSLN1-inside-R	CGAGAGCATGGGTGAGTCCG
SsATF1-inside-F	TCCTTGGCTTGTGCGTTTGC
SsATF1-inside-R	CTCTGTCGTCCTAACCACCA
SsGPA3-inside-F	ATGGGAAACTGTCTTTCTTCCACAGAC
SsGPA3-inside-R	TCACAGAATACCACTATCCTTGAGCG
SsUAC1-inside-F	CCGGCACCTCAGCCATCCTT
SsUAC1-inside-R	AGGTTGTCGTGCTTGCCGAG
SsADR1-inside-F	CAACAACACCGCCGTCCAAG
SsADR1-inside-R	CGTCACGGTCGGCAAATACG
SsSLN1-outside-F	CGGTGCCGGATGATGACTTCT
SsSLN1-outside-R	CGAACACTGACTGGGACAGC
SsATF1-outside-F	CACTTGCCCTTCTGTCGGGT
SsATF1-outside-R	CACCGTGTGGCAGAGGAAGA
SsGPA3-outside-F	GGCCGCCTCGATACTTCAGAG
SsGPA3-outside-R	TGCGGCGCACAGTAAGGTT
SsUAC1-outside-F	GAGACGCGGTTAAGCGGGAA
SsUAC1-outside-R	GGATCGGTCCAGGTTCTTCGG
SsADR1-outside-F	GCCGGAAAGGTAGATGCCCA
SsADR1-outside-R	TCATCCTCTTCGCCATCCACG
The primers for RT-qPCR	
qRT-ACTIN-F	CAGCTCGATGAAGGTCAAGAT
qRT-ACTIN-R	CACATCTGCTGGAAGGTAGAG

qRT-SsGPA3-F	CAAGTACATTCTCTGGCGTTTC
qRT-SsGPA3-R	AGTCGGATGTTGCTCGTATC
qRT-SsUAC1-F	GCACGACAACCTGAGTGTAA
qRT-SsUAC1-R	GCCGTCAAAGAGACCAAAGA
qRT-SsADR1-F	CGTGCTGCTCTACGAAATGC
qRT-SsADR-R	AAGATCCTTGACGCCCGTTT
qRT-SsPRF1-F	CAAGCAGTGTCACCGTTAGA
qRT-SsPRF1-R	GGAGAGCAAGGATGCAAGAT
qRT-SsSLN1-F	TGCATCGTGTTGCCAGAA
qRT-SsSLN1-R	GAGCTGATGGTGACAAACCT
qRT-SsATF1-F	GTGCCAATGCCAAGAACAAG
qRT-SsATF1-R	GTTCTCGGTCGAGTTGGATTT
qRT-SsPTP1-F	GGACATTGATTCGGGTAGCA
qRT-SsPTP1-R	ACCATTGCCGCTCTCATT
qRT-SsPTP2-F	TCTCTACCAAGCCCTCGACT
qRT-SsPTP2-R	GGAATCGATGTTTTCAGCGG
